# Engineering high‐affinity dual targeting cellular nanovesicles for optimised cancer immunotherapy

**DOI:** 10.1002/jev2.12379

**Published:** 2023-11-16

**Authors:** Luyao Zhang, Xu Zhao, Yanan Niu, Xiaoya Ma, Wei Yuan, Jie Ma

**Affiliations:** ^1^ Center of Biotherapy, Beijing Hospital, National Center of Gerontology Institute of Geriatric Medicine Chinese Academy of Medical Sciences Beijing China; ^2^ State Key Laboratory of Molecular Oncology, National Cancer Center/National Clinical Research Center for Cancer/Cancer Hospital Chinese Academy of Medical Sciences and Peking Union Medical College Beijing China

**Keywords:** cellular nanovesicles, high‐affinity consensus, immune checkpoint blockade, PD‐1/PD‐L1, protein engineering, SIRPα/CD47

## Abstract

Dual targeting to immune checkpoints has achieved a better therapeutic efficacy than single targeting due to synergistic extrication of tumour immunity. However, most dual targeting strategies are usually antibody dependent which facing drawbacks of antibodies, such as poor solid tumour penetration and unsatisfied affinity. To meet the challenges, we engineered a cell membrane displaying a fusion protein composed of SIRPα and PD‐1 variants, the high‐affinity consensus (HAC) of wild‐type molecules, and with which prepared nanovesicles (NVs). Through disabling both SIRPα/CD47 and PD‐1/PD‐L1 signalling, HAC NVs significantly preserved the phagocytosis and antitumour effect of macrophages and T cells, respectively. In vivo study revealed that HAC NVs had better tumour penetration than monoclonal antibodies and higher binding affinity to CD47 and PD‐L1 on tumour cells compared with the NVs expressing wild‐type fusion protein. Exhilaratingly, dual‐blockade of CD47 and PD‐L1 with HAC NVs exhibited excellent therapeutic efficacy and biosafety. This study provided a novel biomaterial against tumoural immune escape and more importantly an attractive biomimetic technology of protein delivery for multi‐targeting therapies.

## 1 INTRODUCTION

Immune checkpoint blockade (ICB), especially targeting programmed cell death 1/programmed cell death ligand 1 (PD‐1/PD‐L1) axis has achieved great clinical advances (Gong et al., 2018; Morad et al., 2021). However, current clinical studies have shown that the majority of patients (70%−80%) do not respond to ICB alone, which is due to the neglect of the dynamic and complex cell networks within the tumour microenvironment (TME) (Lei et al., 2020; Molinaro et al., 2016). In addition to T cells, another important cytotoxic cell type observed in TME is macrophage. Through the signal regulatory protein α/cluster of differentiation 47 (SIRPα/CD47) pathway, tumour escape from phagocytic clearance of macrophage (Van Duijn et al., 2022). Therefore, in comparison with targeting one ICB, it should be a more effectively therapeutic strategy to target multi‐immune checkpoints, such as SIRPα/CD47 and PD‐1/PD‐L1 as vital innate and adaptive checkpoints respectively.

When PD‐L1 and CD47 are both over expressed on tumour cells, PD‐L1 sends a ‘don't find me’ signal to the adaptive immune system and CD47 sends a ‘don't eat me’ signal to the innate immune system (Majeti et al., 2009; Tsushima et al., 2007). Interaction of tumour PD‐L1 with PD‐1 on tumour infiltrating lymphocytes (TILs) and tumour CD47 with SIRPα on tumour‐associated macrophages (TAMs) have been recognised as a major mechanism of tumour immune evasion. These pathways are appealing targets with therapeutic implications to synergistically reverse the immune suppressive tumour microenvironment (Chao et al., 2012; Iwai et al., 2017). Recent studies demonstrated that bispecific antibody or fusion protein against both PD‐L1 and CD47 showing better tumour‐cell targeting and stronger therapeutic effect than those of single blockade (Chen, Dominik, et al., 2021; Liu, Liu, et al., 2018). Thus, several studies on CD47/PD‐L1 bispecific antibody are in phase I/II clinical trials (Wang et al., 2023, 2020). However, dual targeting antibody and fusion protein had inherent limitations that curtailed their efficacy in this setting, including poor solid tumour penetration and affinity to ligands respectively (Dahan et al., 2015). To improve the affinity of antibodies, recent studies have genetically modified the binding domains of human PD‐1 and SIRPα, with engineered variants dramatically increasing affinity to their ligands and improving their penetration of targeting tumours (Maute et al., 2015; Weiskopf et al., 2013).

Recent advances in nanotechnology have provided many new opportunities for cancer immunotherapy (Chen et al., 2017; Fang et al., 2018), among which, engineered cellular nanovesicles (NVs) have attracted much attention due to high yield, excellent biosafety and stability, and natural tumour distribution (Andaloussi et al., 2013; Molinaro et al., 2016). Their inherited properties from parental cells exhibit excellent tumour‐specific targeting and biocompatibility compared with commercial liposomes and polymeric nanoparticles (Kamerkar et al., 2017; Karasu et al., 2018). Therefore, it is note‐worthy that NVs have been widely studied to break the limitation of current therapies. For cancer immunotherapy, Rao's group prepared tumour targeting cell vesicles (CVs) by fusion of individual CV components expressing PD‐1 or SIRPα, respectively (Meng et al., 2021). The disadvantage of this method is that the final product expressed uneven distribution of PD‐1 and SIRPα. Therefore, whether the NVs express fusion protein of PD‐1 and SIRPα high‐affinity consensus (HAC) can integrate all the advantages and have synergistic effects on tumour immunomodulation becomes attractive.

**FIGURE 1:**
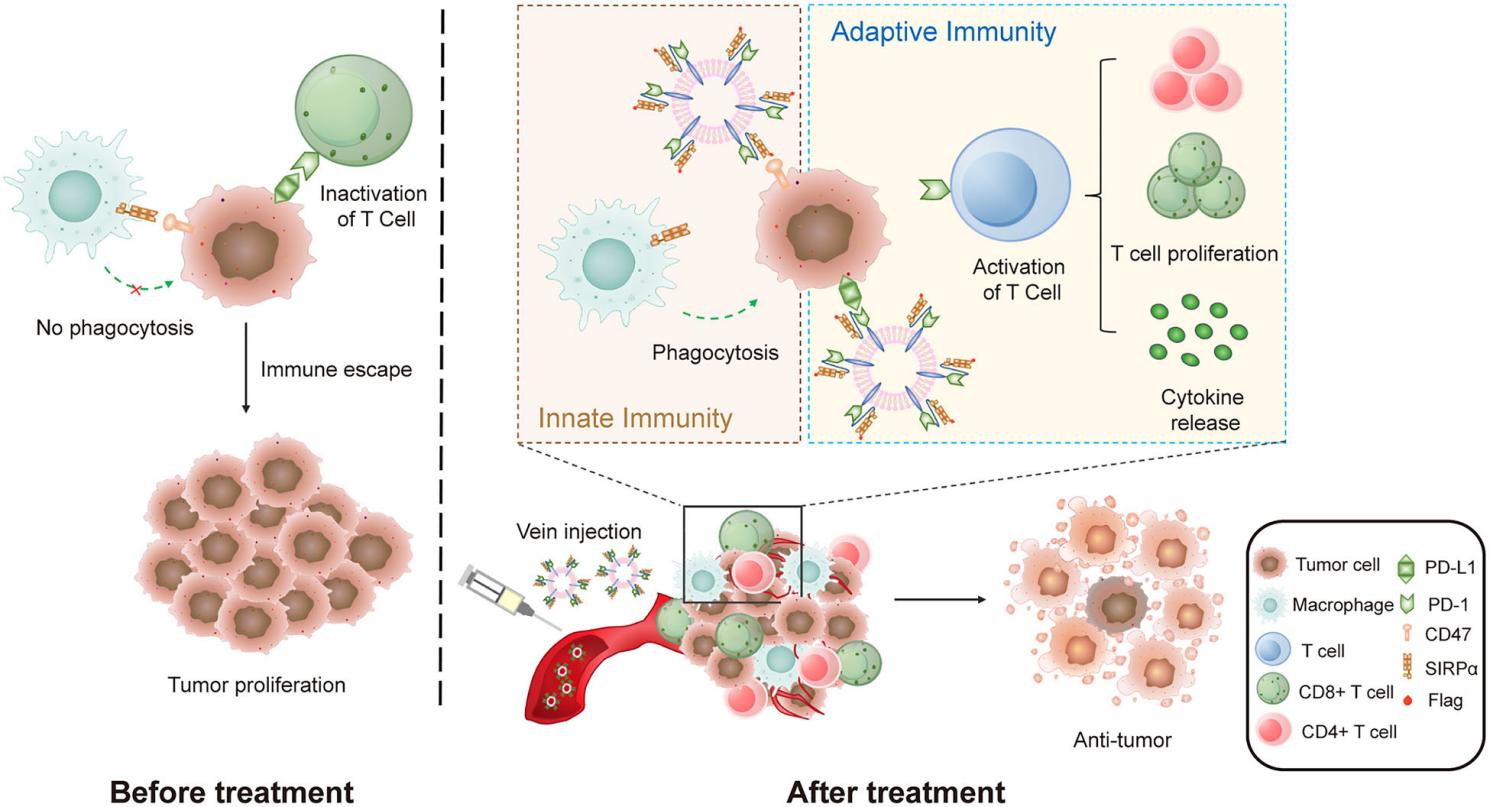
Schematic illustration of fusion HAC NVs for dual‐targeting immune checkpoint blockade therapy. Intravenously injected fusion HAC NVs can distribute to tumour microenvironment and simultaneously block SIRPα/CD47 innate immune pathway and PD‐1/PD‐L1 adaptive immune pathway, promote phagocytosis of macrophages as well as T‐cell antitumour immunity leading to tumour suppression.

In this study, we designed PD‐1/SIRPα fusion HAC NVs, which dual‐target the highly expressed immune checkpoint proteins PD‐L1 and CD47 on tumour cells. The simultaneous blockade of PD‐1/PD‐L1 axis and SIRPα/CD47 axis with fusion HAC NVs achieved promoted antitumour T‐cell responses as well as macrophage phagocytosis (Figure 1). Moreover, the bispecific targeting design of fusion HAC NVs with the variants of antibodies ensured preferable targeting on tumour cells, and resulted in more significant cytostatic effect compared with that of fusion wild‐type (WT) NVs or dual targeting antibody (Atezolizumab and Magrolimab). It is a valuable attempt to develop PD‐1/SIRPα fusion HAC NVs as direct immunotherapeutic agents for tumour ICB therapy.

## 2 MATERIALS AND METHODS

### 2.1 Materials, cells and animals

Pierce BCA Protein Assay Kit, CellTracker™ Red and CellTracker™ Green were purchased from Thermo Fisher Scientific (Massachusetts, USA). RIPA lysis buffer, penicillin‐streptomycin were obtained from Solarbio (Beijing, China). Anti‐Flag‐agarose beads and Flag peptide were obtained from Bimake (Shanghai, China). UltraFection 3.0, Human IFN‐γ Elisa Kit, Human IL‐6 Elisa Kit and Human IL‐8 Elisa Kit were purchased from 4A BIOTECH (Beijing, China). Recombinant Human IFN‐gamma was purchased from PeproTech (New Jersey, USA). PC5.5‐anti‐Human‐CD45, PE‐anti‐Human CD4 and FITC‐anti‐Human CD8 antibodies were purchased from BD Pharmingen (New Jersey, USA). Eastep Super® Total RNA Extraction Kit was purchased by Promega (Madison, USA). Annexin V, 633 Apoptosis Detection Kit and Calcein‐AM/PI Double Staining Kit was purchased from Dojindo Laboratories (Tokyo, Japan). One‐step TUNEL Apoptosis Assay Kit and Hoechst 33342 were obtained from Beyotime (Shanghai, China). Atezolizumab was obtained from Selleck (Texas, USA), and Magrolimab was bought from AtaGenix (Wuhan, China). Anti‐Perforin, anti‐Granzyme B, anti‐Mannose Receptor, anti‐CD68, anti‐CD81 and anti‐calnexin antibodies were purchased from Abcam (Cambridge, UK). Anti‐CD9, anti‐CD63, anti‐PD‐L1, anti‐CD47 and anti‐β‐actin antibodies were purchased from Proteintech (Wuhan, China). Anti‐flag was obtained from Cell Signaling Technology (Boston, USA). EdU Imaging Kits (Cy3) was purchased from APExBIO (Houston, USA). PrimeScript™ RT reagent kit with gDNA Eraser and TB Green Premix Ex Taq™ were obtained from Takara (Japan).

All cells were preserved by Cancer Hospital Chinese Academy of Medical Sciences (Beijing, China). HEK293 cells, MDA‐MB‐231 cells and A549 cells were cultivated in Dulbecco's modified eagle medium (DMEM; Sigma, USA), Leibovitz's L‐15 medium (Gibco, USA) and Ham's F‐12K medium (Gibco, USA) containing 10% fetal bovine serum (FBS) and 1% penicillin‐streptomycin, respectively. The cells were incubated in a 37°C‐incubator containing 5% CO_2_.

Female SPF BALB/c‐nude mice (5 weeks old, 18–19 g) were purchased from Beijing Huafukang Bioscience Co. Ltd. All animals were bred in a specific pathogen‐free environment. All animal procedures were in accordance with all relevant guidelines of the Institutional Animal Care and Use Committee of Cancer Hospital of Chinese Academy of Medical Sciences (No. NCC2021A278).

### 2.2 Generation of engineered nanovesicles

We transfected HEK293 cells with an expression plasmid to construct engineered cells. The cells were collected and washed with PBS via centrifugation, and the supernatant was discarded. The cell pellet was gently resuspended in an appropriate amount of PBS containing PMSF (final concentration of 1 mM). Ultrasonication was performed for 1 min (2 s on, 2 s off, 100 w) using an ultrasonic cell pulveriser (Scientz‐IID, China). The supernatant was collected for further centrifugation at 20,000 g for 30 min at 4°C to remove the nucleus and intracellular membrane, and then extruded and filtered with a 0.22 μm filter to acquire cell‐membrane nanovesicles. The resulting nanovesicles were quantified using a Pierce BCA Protein Assay Kit and dissolved in PBS and stored at 4°C for further study.

### 2.3 Characterisation of NVs

The size distributions of Blank, Flag‐Fusion WT and Flag‐fusion HAC NVs were detected using a Nanoparticle Tracking Analysis (Malvern NS300, UK). We collected potential of vesicles by dynamic light scattering (DLS) (Malvern Zetasizer Nano ZS90, UK). All measurements were performed three times (*n* = 3) at 25°C and the results were counted as mean ± SD. All samples were respectively dropped on copper grids and stained with 1% uranyl acetate. The morphology of vesicles was examined using a transmission electron microscope (TEM) (JEM‐1400 plus, Japan).

### 2.4 Co‐immunoprecipitation (Co‐IP)

Coimmunoprecipitation (Co‐IP) assay was employed to examine the interaction of tumour cells with the fusion WT NVs. In brief, 100 μg/mL Flag‐fusion WT or Flag‐fusion HAC NVs were added and incubated with tumour cells (10 cm dish) for 12 h. After that the cells were washed three times with PBS to remove the unbound NVs. And then, the cells were lysed in 1 mL RIPA lysis buffer containing phosphatase inhibitor cocktail. The cell lysis was then centrifuged at 15,000 × *g* for 10 min at 4°C. After that the lysates were incubated with 15 μL anti‐Flag‐agarose beads for 2 h at 4°C with gentle rotation. Then the beads were then washed gently with ice‐cold RIPA buffer three times. The bound proteins were eluted by Flag peptide and subjected to 10% SDS‐PAGE and analysed by western immunoblot using the indicated antibodies. Data were collected from at least three independent experiments.

### 2.5 Western blot

Equal amounts (20–50 μg) of proteins were size‐fractionated by 7.5%−10% (w/v) SDS/PAGE. The antibodies used were anti‐CD9, anti‐CD81, anti‐TSG101, anti‐calnexin, anti‐PD‐L1, anti‐CD47, anti‐β‐actin and anti‐Flag.

### 2.6 FACS analysis

For antibody competition assay, 1 × 10^6^ MDA‐MB‐231 or A549 cells were harvested and incubated with 200 μg/mL Blank, Flag‐Fusion WT and Flag‐fusion HAC NVs or 20 μg/mL Atezolizumab and Magrolimab for 30 min, respectively. After that the cells were washed three times with PBS to remove the unbound NVs. Then, adding fluorescent‐labelled specific antibody (FITC‐anti‐PD‐L1, PC7‐anti‐CD47), the incubation was continued for 30 min. All samples were analysed by Flow Cytometry (Beckman Coulter, USA).

For tumour infiltrated T cells detection, the percentage of CD8^+^/CD45^+^ T and CD4^+^/CD45^+^ T cells were evaluated using flow cytometry. Tumour tissues were efficiently dissociated to collect single‐cell suspensions with a GentleMACS Dissociator (Miltenyi Biotec, Germany) according to the manufacturer's instructions. Briefly, we firstly removed fibrous and necrotic areas of tumour samples and cut them into small pieces. Subsequently, the tumour pieces were transferred into gentleMACS C Tubes containing the enzyme mixture, respectively. The C Tubes were loaded onto the gentleMACS Dissociator carefully, then ran the selected program. Using a 70 μm mesh cell strainer to collect filtered cell suspensions into a 50 mL centrifuge tube. The cell suspensions were washed with RPMI 1640 medium and PBS at 300 × *g* for 7 min, respectively. The following immune cells were stained with PC5.5‐anti‐Human‐CD45, PE‐anti‐Human CD4 and FITC‐anti‐Human CD8 antibodies for 20 min. All samples were analysed by Flow Cytometry (Beckman Coulter, USA).

### 2.7 CCK‐8 assay

Equal numbers of MDA‐MB‐231 cells (5 × 10^3^/well) were seeded into a 96‐well plate 24 h before experimentation. Cells were treated with 100 μg Blank, Flag‐Fusion WT and Flag‐fusion HAC NVs for 12 h, 24 h, 48 h, respectively. After treatment, CCK‐8 was added to the 96‐well plate and incubated at 37°C for 1 h. The absorbance of each sample was read at 450 nm.

### 2.8 EdU incorporation assay

MDA‐MB‐231 cells were treated with 100 μg Blank, Flag‐Fusion WT, or Flag‐Fusion HAC NVs for 48 h. For EdU analysis, MDA‐MB‐231 cells were cultured with 10 μM EdU for 2 h and then treated with 4% paraformaldehyde for fixation. After permeabilisation, EdU Imaging Kits (Cy3) was used according to the manufacturer's instructions. Hoechst 33342 solution was used to counterstain cell nuclei. Proliferative cells were determined under the confocal microscope (PerkinElmer ULTRAVIEW VOX, USA).

### 2.9 Live/dead cell staining

1 × 10^5^ MDA‐MB‐231 cells were seeded in a 24‐well plate. Afterward, 100 μg Blank, Flag‐Fusion WT and Flag‐fusion HAC NVs were added to the plate. After incubation for 24 h, the cells were washed gently with PBS three times. After that, the cells were stained with Calcein‐AM/PI for 20 min at dark. Using a fluorescence microscope to investigate the relative viability of MDA‐MB‐231 cells after different treatments (Leica, Germany).

### 2.10 In vitro apoptosis analysis

To further investigate the antitumour activity of nanovesicles in vitro, we carried out Annexin/PI staining assay. 2 × 10^5^ MDA‐MB‐231 cells were seeded in 6‐well plates and incubated for 24 h to attach. Then the cells were incubated with 100 μg Blank, Flag‐Fusion WT and Flag‐fusion HAC NVs. After 48 h, the cells were collected and stained following the manufacturer's instructions. Finally, apoptosis in different treatments were evaluated by flow cytometry (Beckman Coulter, USA).

### 2.11 Wound‐healing assay

We used the scratching method to study the effect of Flag‐fusion HAC NVs on cell migration. 2 × 10^5^ MDA‐MB‐231 cells were seeded in 6‐well plates and cultured until 90% confluent. After that, cells were scratched using a 200 μL pipette tips and then treated with NVs. Finally, the images of scratched areas were obtained at the appointed times using an inverted microscope. Besides that, A549 cells were pretreated with recombinant human interferon‐gamma (100 ng/mL) for 12 h, then detected the change of the wound width as above method.

### 2.12 In vitro analysis of the phagocytosis of cancer cells by macrophages

For fluorescence microscopic analysis, MDA‐MB‐231 cells were first incubated with indicated concentrations of Blank, Flag‐fusion WT or Flag‐fusion HAC NVs. After that, THP‐1 derived macrophages and MDA‐MB‐231 cells were stained with CellTracker™ Red and CellTracker™ Green, respectively. Fluorescence labelled THP‐1 derived macrophages and MDA‐MB‐231 cells were then co‐cultured for 24 h. For FACS analysis, the MDA‐MB‐231 cells phagocytosis by THP‐1 derived macrophages cells was evaluated as the percentage of green fluorescent positive macrophages. And for fluorescence microscopic analysis, phagocytosis was measured as green fluorescence signalling related to the formation of phagosomes in macrophages.

### 2.13 In vitro analysis of the T‐cell activation

1 × 10^7^ PBMC were cultured in T cell specific medium for 72 h, and activated in the plate coating anti‐CD3/anti‐CD28 antibodies (anti‐CD3 2 μg/mL; anti‐CD28 2 μg/mL, Thermo Fisher Scientific) for 48 h. MDA‐MB‐231 cells (1 × 10^6^ cells/mL) were first incubated with Blank, Flag‐fusion WT or Flag‐fusion HAC NVs at the concentration of 100 μg/mL and then co‐cultured with the resulting activated CD8^+^ T cells at the ratio of 1:1 for 48 h, after which the supernatants were collected for measurement of IFN‐γ production by enzyme‐linked immunosorbent assay (ELISA).

### 2.14 Nanovesicles direct effect on immune cells

Activated T cells and macrophages were cultured as mentioned above, following by incubating with Blank, Flag‐fusion WT or Flag‐fusion HAC NVs at the concentration of 100 μg/mL for 24 h, respectively. The expression levels of function related proteins in activated T cells and macrophages were detected by qPCR.

### 2.15 Penetration in multicellular MDA‐MB‐231 tumour spheroids

MDA‐MB‐231 cells were seeded into a 96‐well ultralow attachment plate at 3 × 10^3^ cells per well and grew in a 37°C incubator with 5% CO_2_. The medium was replaced every two days with fresh culture medium for a week to form multicellular spheroids with an appropriate size. Afterward, the tumour spheroids were incubated with the same amount of DiO‐labelled different vesicles for 6 h, and visualised permeation of tumour spheroids on Confocal Laser Scanning Microscopy by sequential scan (PerkinElmer ULTRAVIEW VOX, USA).

### 2.16 Biodistribution of NVs in tumour‐bearing mice

To examine the biodistribution of Flag‐Fusion WT and Flag‐fusion HAC NVs in MDA‐MB‐231 tumour‐bearing mice, Flag‐Fusion WT and Flag‐fusion HAC NVs were labelled with DiR. When the volume of the orthotopic MDA‐MB‐321 tumour reached 300−400 mm^3^, mice were randomly divided into three groups. PBS, DiR‐labelled Flag‐Fusion WT and Flag‐fusion HAC NVs were intravenously injected into mice, respectively (10 mg/kg). After 24 h post‐administration, the mice were sacrificed and major tissues (heart, liver, spleen, lung, kidney and tumour) were harvested, the fluorescent distribution images were captured using an IVISs Lumina system (PerkinElmer, USA).

### 2.17 Therapeutic efficacy of NVs in vivo

Approximately 2 × 10^6^ MDA‐MB‐231 cells were implanted into the right breast pads of BALB/c nude mice. When the tumour size approximately 40 mm^3^, mice were randomly divided into four groups (*n* = 6). (1) Blank NVs (10 mg/kg); (2) Flag‐Fusion WT NVs (10 mg/kg); (3) Flag‐Fusion HAC NVs (10 mg/kg); (4) Atezolizumab + Magrolimab (1 mg/kg). Approximately 5×10^6^ PBMC were intravenously administrated once a week, and different treatments via the tail intravenous injection three times a week. Tumour volumes and body weights were measured and recorded every 2 days. The tumour volumes were calculated as V = (length×width^2^) / 2. On the 15th day of treatment, all mice from each group were sacrificed and the tumours were resected, taken photographs and weighed. Major organs (lung, heart, spleen, liver and kidney) and blood were also harvested.

### 2.18 Assessing systemic toxicity

The whole blood was collected through mice's eyeball removal in each group before euthanasia for the routine blood examination. To evaluate the level of liver and renal function after different treatments, we determined the levels of alanine transaminase (ALT), aspartate transaminase (AST), creatinine (CRE) and blood urea nitrogen (BUN) in the plasma. Meanwhile, the major organs were collected and fixed with 4% paraformaldehyde for 48 h, stained paraffin‐embedded tissue with haematoxylin‐eosin (H&E) for histopathological analyses. The sections were observed with a microscope (Leica, Germany).

### 2.19 Immunohistochemical and immunofluorescence staining of tissue sections

Sectioning and immunohistochemical (IHC) staining of formalin fixed, paraffin‐embedded mice xenograft tumour specimens were performed by the standard protocols. All sections were 5 mm thick. Briefly, sections were deparaffinised through xylenes and graded ethanol, and antigen retrieval was performed in Tris/EDTA buffer at pH 9.0. For cell apoptosis, tumour tissues were evaluated using the one‐step TUNEL kit according to the manufacturer's instructions. For multiple immunofluorescence staining, PANO 4‐plex IHC kit (PANOVUE) was used according to the manufacturer's instructions. The samples were then observed under a fluorescence microscope. CD4, CD8 and CD45 antibodies were purchased from Proteintech (Wuhan, China), and CD68, CD86 and CD206 antibodies were purchased from Abcam (Cambridge, UK).

### 2.20 Quantitative Real‐time PCR analyses

Total RNAs of tissues were extracted using Eastep Super® Total RNA Extraction Kit, and quantified the concentration of tissue RNA via NanoDrop One (Thermo Scientific, USA). One microgram of total RNA was reverse transcribed into cDNA using a PrimeScript™ RT reagent kit with gDNA Eraser. Then RT‐qPCR was performed with TB Green Premix Ex Taq™ (Applied Biosystems 7500 Fast Real‐Time PCR System, Applied Biosystems). The primer sequences were as follows:

**Table:** 

	*Forward*	*Reverse*
*CD4*	*TGCCTCAGTATGCTGGCTCT*	*GAGACCTTTGCCTCCTTGTTC*
*CD8A*	*TCCTCCTATACCTCTCCCAAAAC*	*GGAAGACCGGCACGAAGTG*
*IFNG*	*TCGGTAACTGACTTGAATGTCCA*	*TCGCTTCCCTGTTTTAGCTGC*
*PRF1*	*GACTGCCTGACTGTCGAGG*	*TCCCGGTAGGTTTGGTGGAA*
*GZMB*	*TACCATTGAGTTGTGCGTGGG*	*GCCATTGTTTCGTCCATAGGAGA*
*CD68*	*TGGGGCAGAGCTTCAGTTG*	*TGGGGCAGGAGAAACTTTGC*
*CD86*	*CTGCTCATCTATACACGGTTACC*	*GGAAACGTCGTACAGTTCTGTG*
*MRC1*	*GGGTTGCTATCACTCTCTATGC*	*TTTCTTGTCTGTTGCCGTAGTT*
*TGFB1*	*GGCCAGATCCTGTCCAAGC*	*GTGGGTTTCCACCATTAGCAC*
*IL6*	*ACTCACCTCTTCAGAACGAATTG*	*CCATCTTTGGAAGGTTCAGGTTG*
*IL10*	*TCAAGGCGCATGTGAACTCC*	*GATGTCAAACTCACTCATGGCT*
*TNF*	*CCTCTCTCTAATCAGCCCTCTG*	*GAGGACCTGGGAGTAGATGAG*
*CXCL8*	*TTTTGCCAAGGAGTGCTAAAGA*	*AACCCTCTGCACCCAGTTTTC*
*ACTIN*	*CATGTACGTTGCTATCCAGGC*	*CTCCTTAATGTCACGCACGAT*
*ELMO1*	*GGAGCAGGTTATGAGAGCACT*	*GGGCGGGACTGGAAATCTTC*
*DOCK1*	*ACCGAGGTTACACGTTACGAA*	*TCGGAGTGTCGTGGTGACTT*
*RAC1*	*ATGTCCGTGCAAAGTGGTATC*	*CTCGGATCGCTTCGTCAAACA*
*GAPDH*	*GGAGCGAGATCCCTCCAAAAT*	*GGCTGTTGTCATACTTCTCATGG*

### 2.21 Statistical analysis

The data represent the means ± SD of at least two independent experiments. Statistical analyses were performed by GraphPad Prism 8. The differences between two groups were assessed by unpaired two‐tailed Student's *t* tests. Multi‐group comparisons were analysed by one‐way or two‐way ANOVA with Tukey's post hoc test. *p* < 0.05 was considered statistically significant (*p < 0.05, **p < 0.01 and ***p < 0.001).

## 3 RESULTS

### 3.1 Dual target cellular nanovesicles produced from engineered high‐affinity consensus expressing cell

**FIGURE 2:**
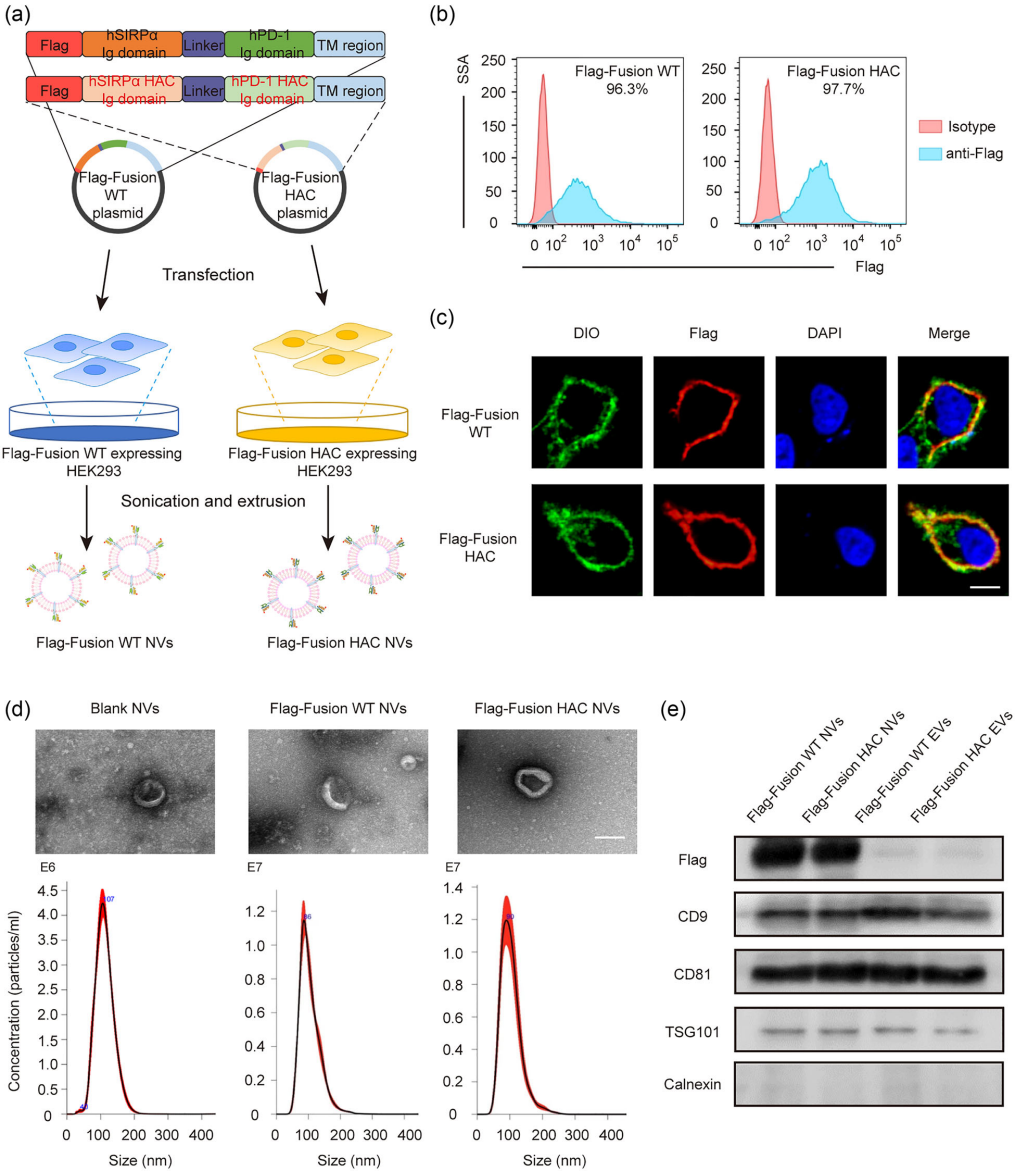
High‐affinity consensus expressed cell construction and cellular nanovesicles preparation and characterisation. (a) Schematic illustration of plasmids construction and cellular nanovesicles preparation of SIRPα‐PD‐1 wild‐type (Flag‐Fusion WT) and HAC (Flag‐Fusion HAC) fusion. (b) The expression level of Flag‐Fusion WT and Flag‐Fusion HAC was confirmed by flow cytometry in HEK293. (c) Representative immunofluorescence images of Flag‐Fusion WT and Flag‐Fusion HAC expression (Red) in HEK293 cells. The cell membranes were stained with DiO (Green). The nuclei were stained with DAPI (Blue). Scale bar: 10 μm. (d) Morphology characterization (top) and the size distribution (bottom) of Blank, Flag‐Fusion WT, and Flag‐Fusion HAC NVs. Scale bar: 100 nm. (e) Western blot analysis of plasma membrane markers CD9, CD81, TSG101 and endoplasmic reticulum marker Calnexin in Flag‐Fusion WT EVs, Flag‐Fusion HAC EVs, Flag‐Fusion WT NVs and Flag‐Fusion HAC NVs.

Given that mutations of specific amino acids on the extracellular immunoglobulin (Ig) domain of PD‐1 and SIRPα have been found to enhance their affinity for ligands dramatically in vitro (Maute et al., 2015; Weiskopf et al., 2013). We thus designed plasmids that co‐expressing the wild‐type PD‐1 and SIRPα Ig domain (Flag‐Fusion WT) or its high‐affinity consensus (Flag‐Fusion HAC) with amino acid mutations at specific sites, and then transfected them into HEK293 cells, respectively (Table S1 and Figure 2a). Flow cytometry showed that the expression efficiency of these two engineered plasmids achieved more than 95% (Figure 2b). Confocal imaging also visually revealed that the expressed fusion proteins located on the plasma membrane (Figure 2c). These results showed that we successfully constructed engineered cells with high expression of the fusion proteins.

Next, Flag‐Fusion WT and Flag‐Fusion HAC cellular nanovesicles (NVs) were harvested and prepared from cultured HEK293 cells by sonication and extrusion (Figure 2a) (Andaloussi et al., 2013). Transmission electron microscopy (TEM) visualisation and Nanoparticle Tracking Analysis (NTA) suggested that the particle size of fusion WT NVs were approximately 100 nm with the average surface ζ potential of −12 mV, which had the similar morphological characteristics of spherical bilayered proteolipids as the blank NVs (Figures 2d and S1). Moreover, we isolated naturally produced EVs of genetically engineered cells and compared them with NVs. Western blot revealed that classical EV protein markers, including CD9, CD81 and TSG101, were detected in fusion WT NVs and fusion HAC NVs, whereas, EV exclusion marker, calnexin, was not detected in the NVs (Figure 2e). However, the content of fusion WT or fusion HAC proteins in NVs was much higher than that in EVs (Figure 2e). These results revealed no significant difference in protein marker was found between naturally produced EVs and NVs, but fusion proteins expressed in plasma membrane were highly abundant on NVs. Overall, the above results indicated that the Flag‐Fusion WT and Flag‐Fusion HAC NVs could be used as an efficient PD‐1 and SIRPα variable immunoglobulin cell membrane‐derived biomimetic delivery vehicle.

### 3.2 HAC NVs interact efficiently with the ligands PD‐L1 and CD47

**FIGURE 3:**
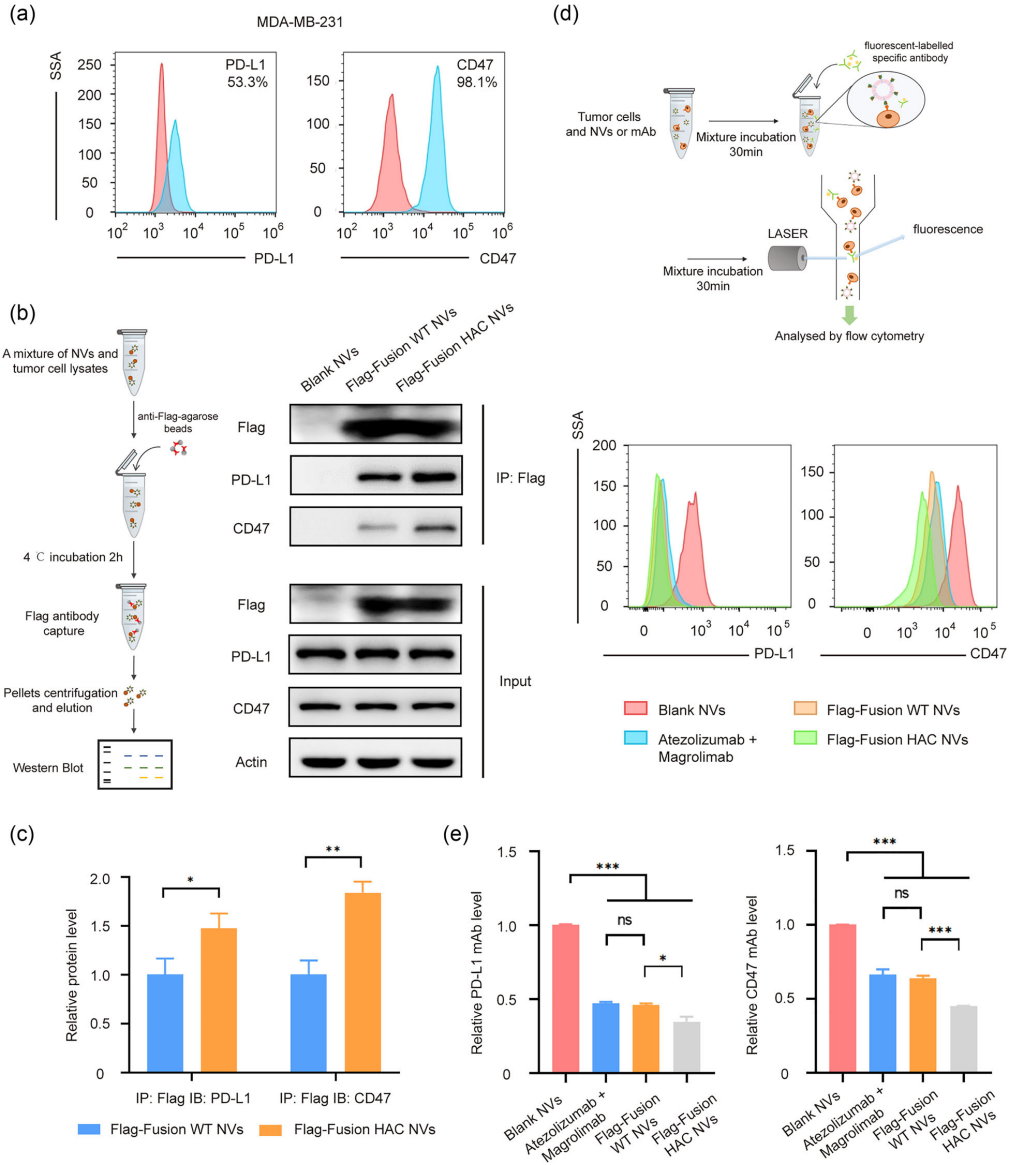
Interaction efficiency of HAC NVs with the ligands of PD‐L1 and CD47. (a) The expression level of PD‐L1 and CD47 was measured by flow cytometry in MDA‐MB‐231 cell (Red: Isotype matched antibody; Blue: anti‐PD‐L1 or anti‐CD47 antibody). (b) Schematic illustration (left) and representative western blot analysis (right) of coimmunoprecipitation of Blank, Flag‐Fusion WT or Flag‐Fusion HAC NVs interacting with PD‐L1 and CD47. (c) Quantitative analysis of immunoprecipitation in (b) (*n* = 3, *p < 0.05, **p < 0.01). (d) Schematic illustration of NVs and mAb competitive binding assay (top). Binding and blocking efficiency of Blank, Flag‐Fusion WT NVs, Flag‐Fusion HAC NVs or combination of Atezolizumab and Magrolimab to PD‐L1 and CD47 were measured by flow cytometry in MDA‐MB‐231 cell (bottom). (e) Quantitative analysis of PD‐L1 or CD47 competing binding efficiency against NVs or combination of mAb in (d) (*n* = 3, *p < 0.05, ***p < 0.001).

We then explored the affinity of fusion HAC NVs and their ligands PD‐L1 and CD47 on tumour cells. We firstly performed flow cytometry to detect the expression levels of PD‐L1 and CD47 on triple‐negative breast cancer (TNBC) cells MDA‐MB‐231 and 100 ng/mL IFN‐γ pre‐treated lung adenocarcinoma cells A549. We verified that PD‐1 and CD47 were highly expressed on these cells as previously reported (Bian et al., 2022; Lee et al., 2019) (Figures 3a and S2). Next, we employed coimmunoprecipitation (Co‐IP) assay to examine the interaction of tumour cells with the engineered fusion WT NVs. After incubation of 100 μg/mL fusion WT NVs or fusion HAC NVs with MDA‐MB‐231 cells for 12 h, the cells were harvested. Flag primary antibody was used to pull down the Flag‐SIRPα‐PD‐1 on the NVs. Remarkably, PD‐L1 or CD47 was pulled down together with Flag‐SIRPα‐PD‐1 by the Flag antibody (Figures 3b and S3A). We found that both PD‐L1 and CD47 molecules on MDA‐MB‐231 cells had interaction with fusion WT NVs, and the binding efficiency of the fusion HAC NVs was obviously increased than the fusion WT NVs (Figure 3b,c). Meanwhile, another result showed that the fusion HAC NVs had the increased binding features to A549 cells treated with IFN‐γ, similar to MDA‐MB‐231 cells (Figure S3B,C).

To further confirm the binding affinity of fusion HAC NVs to ligands on tumour cells, competitive binding assay were performed. Briefly, 200 μg/mL NVs or 20 μg/mL Atezolizumab and Magrolimab were thoroughly mixed with 1 × 10^6^ MDA‐MB‐231 cells for 30 min to bind PD‐L1 and CD47 expressed on tumour cells. After removing the unbound NVs, the same amount of fluorescent‐labelled PD‐L1 or CD47 antibody was added for another 30 min to interact with the remaining epitopes after NVs or blocking antibodies binding. We found that the fluorescent signal of PD‐L1 on cells binding to blocking antibodies, fusion WT NVs and fusion HAC NVs was reduced obviously compared with blank NVs group, especially the interaction between fusion HAC NVs and PD‐L1, with ∼65%, ∼12% and ∼11% lower than blank NVs, fusion WT NVs and blocking antibodies groups, respectively (Figure 3d,e). In addition, the binding levels of CD47 epitopes to specific fluorescent antibodies were also significantly reduced in cells binding to fusion HAC NVs, with ∼55%, ∼33% and ∼37% lower than blank NVs, fusion WT NVs and blocking antibodies groups, respectively (Figure 3d,e). The strong interaction between HAC NVs and tumour cells was also found on A549 stimulated by 100 ng/mL IFN‐γ. Specifically, the binding levels of fluorescent PD‐L1 antibody with cells binding to fusion HAC NVs was diminished ∼55%, and that of fluorescent CD47 achieved ∼75% reduction compared with blank NVs group (Figure S4A‐B). These results suggested that the fusion WT and fusion HAC NVs could interact with immune checkpoint proteins on tumour cells in vitro, and fusion HAC NVs showed more efficiently binding to the ligands compared to the fusion WT NVs and epitopes specific blocking antibodies.

Furthermore, we investigated the expression of PD‐L1 and CD47 in Human Protein Atlas (HPA) database, and found that the expressions of CD47 in breast tumour cells (MCF‐7, MDA‐MB‐231) were significantly higher than that in normal cells (HEK293, MCF‐10A), while PD‐L1 was only significantly expressed in MDA‐MB‐231 cells with high malignance (Figure S5A). To test the binding and uptake ability of NVs by the tumour cells with different malignancy, fusion HAC NVs labelling green fluorescent dye were incubated with HEK293, MCF‐7, MDA‐MB‐231 cells for 6 h, respectively, and then imaged with fluorescence microscopy. We found significant green fluorescence in the periphery and inside of MDA‐MB‐231 cells, and the proportion of NVs positive cells was ∼6‐fold and ∼1.5‐fold than that of HEK293 and MCF‐7 cells, respectively (Figure S5B,C). These results indicated that fusion HAC NVs anchored to ligands on the surface and could be internalised into tumour cells. The recruitment of NVs was more obvious in tumours with high malignancy.

### 3.3 HAC NVs inhibit immune‐independent tumour cell growth and migration

**FIGURE 4:**
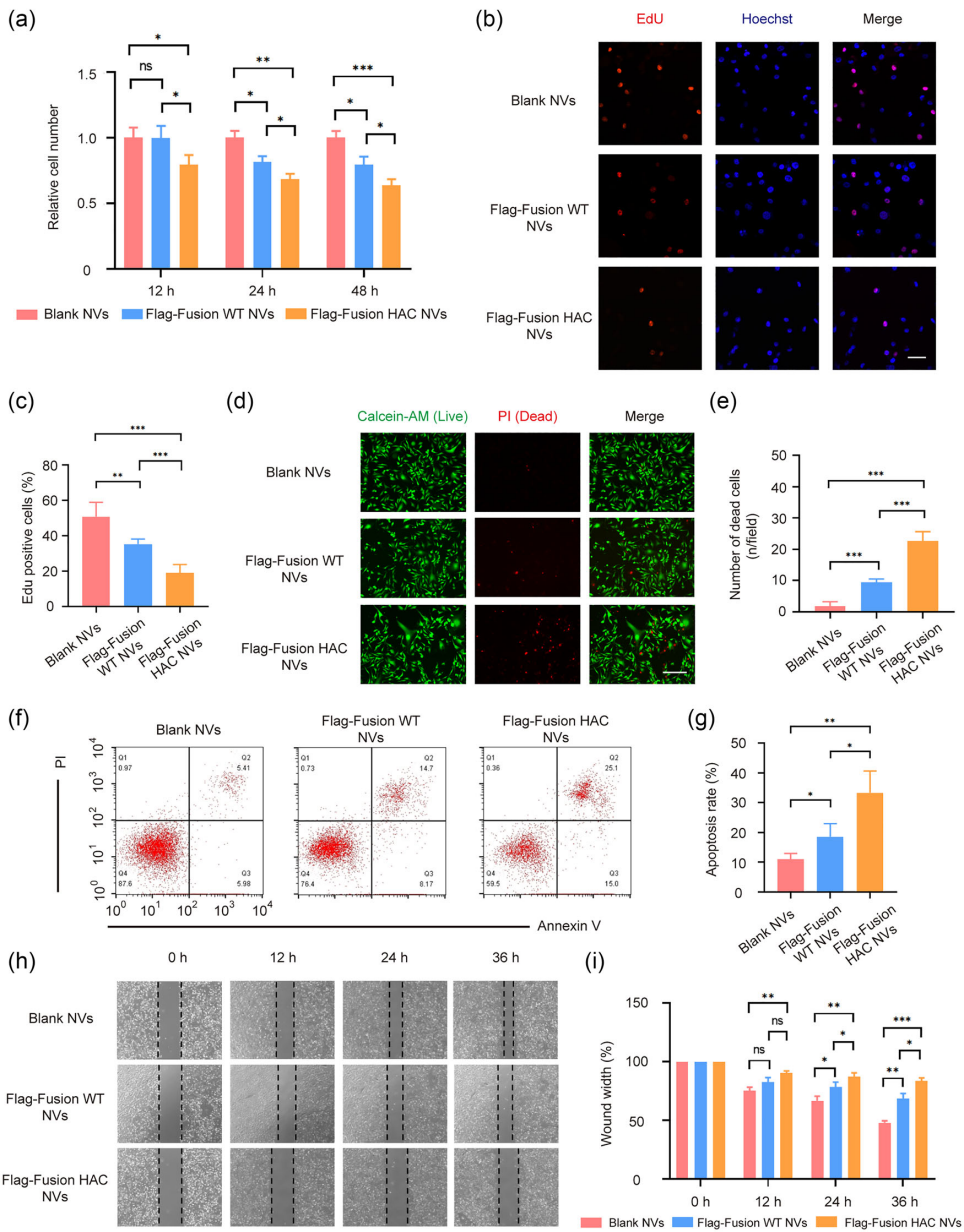
HAC NVs inhibit immune‐independent tumour cell growth and migration. (a) Cell viability for 12, 24 and 48 h after MDA‐MB‐231 cells were treated with 100 μg Blank, Flag‐Fusion WT and Flag‐Fusion HAC NVs, respectively (*n* = 3, *p < 0.05, **p < 0.01 and ***p < 0.001). (b) Fluorescence images of dividing MDA‐MB‐231 cells in each group (Red: Edu staining; Blue: Hoechst staining). Scale bar: 50 nm. (c) Quantification of proportion of dividing cells in (b) (*n* = 5, **p < 0.01, ***p < 0.001). (d) Fluorescence images of survival and death of MDA‐MB‐231 cells in each group (Green: Calcein‐AM staining; Red: PI staining). Scale bar: 100 nm. (e) Quantification of proportion of dead cells in (d) (*n* = 3, ***p < 0.001). (f) Representative images of apoptotic status of MDA‐MB‐231 cells treated with 100 μg Blank, Flag‐Fusion WT or Flag‐Fusion HAC NVs for 48 h. (g) Quantitative analysis of cell apoptosis in (f) (*n* = 3, *p < 0.05, **p < 0.01). (h) Wound healing assay of MDA‐MB‐231 cells treated with different NVs. Representative images of cell migration were shown. (i) Quantitative analysis of wound closure in (h) (*n* = 3, *p < 0.05, **p < 0.01 and ***p < 0.001).

Several studies reported that PD‐L1 and CD47 also played a vital role in regulating immune‐unrelated biological processes in tumour cells, moreover, antagonising PD‐L1 or CD47 could inhibit the tumour cell proliferation and migration and induce apoptosis (Azarbarzin et al., 2021; Clark et al., 2016; Hu et al., 2020; Kong et al., 2020; Zhang et al., 2020). Based on this, we investigated whether the fusion HAC NVs had function to immune‐independent antitumour activities. At first, we performed a CCK‐8 cell proliferation assay and demonstrated that MDA‐MB‐231 cell viability was reduced after fusion WT NVs or fusion HAC NVs treatment, in comparison with blank NVs treated group (Figure 4a). Similarly, an EdU assay showed the division percentage of MDA‐MB‐231 cells in fusion WT NVs treated group and fusion HAC NVs treated group was ∼1.4‐fold and ∼2.7‐fold less, respectively, than that of blank NVs (Figure 4b,c). We then stained live cells with Calcein‐AM and dead cells with PI, and found that fusion HAC NVs treated group showed significant cell death, which was ∼12.5‐fold and ∼2.4‐fold more than that of blank NVs and fusion WT NVs treated group, respectively (Figure 4d,e). Flow cytometry analysis displayed obvious apoptosis of MDA‐MB‐231 cells following treatment with blank NVs, fusion WT NVs and fusion HAC NVs for 48 h, respectively. In fusion HAC NVs group, the percentage of both early and late apoptotic cells was increased ∼2.5‐fold over the blank NVs (Figure 4f,g). Moreover, we performed a wound‐healing assay to evaluate the effect of fusion HAC NVs on the tumour cell migration in vitro. After incubation for 36 h, the wound‐closure rate of MDA‐MB‐231 cells with fusion HAC NVs treatment was ∼1.7‐fold and ∼1.2‐fold slower than blank NVs and fusion WT NVs treated groups, respectively (Figure 4h,i). The slower rate of wound healing after treatment with fusion HAC NVs was also found in A549 cells stimulated with IFN‐γ (Figure S6A,B). These results, collectively, suggested that the fusion HAC NVs inhibit immune‐independent tumour cell proliferation, apoptosis and migration.

### 3.4 HAC NVs block CD47 and PD‐L1 checkpoints to promote antitumour immunity in vitro

**FIGURE 5:**
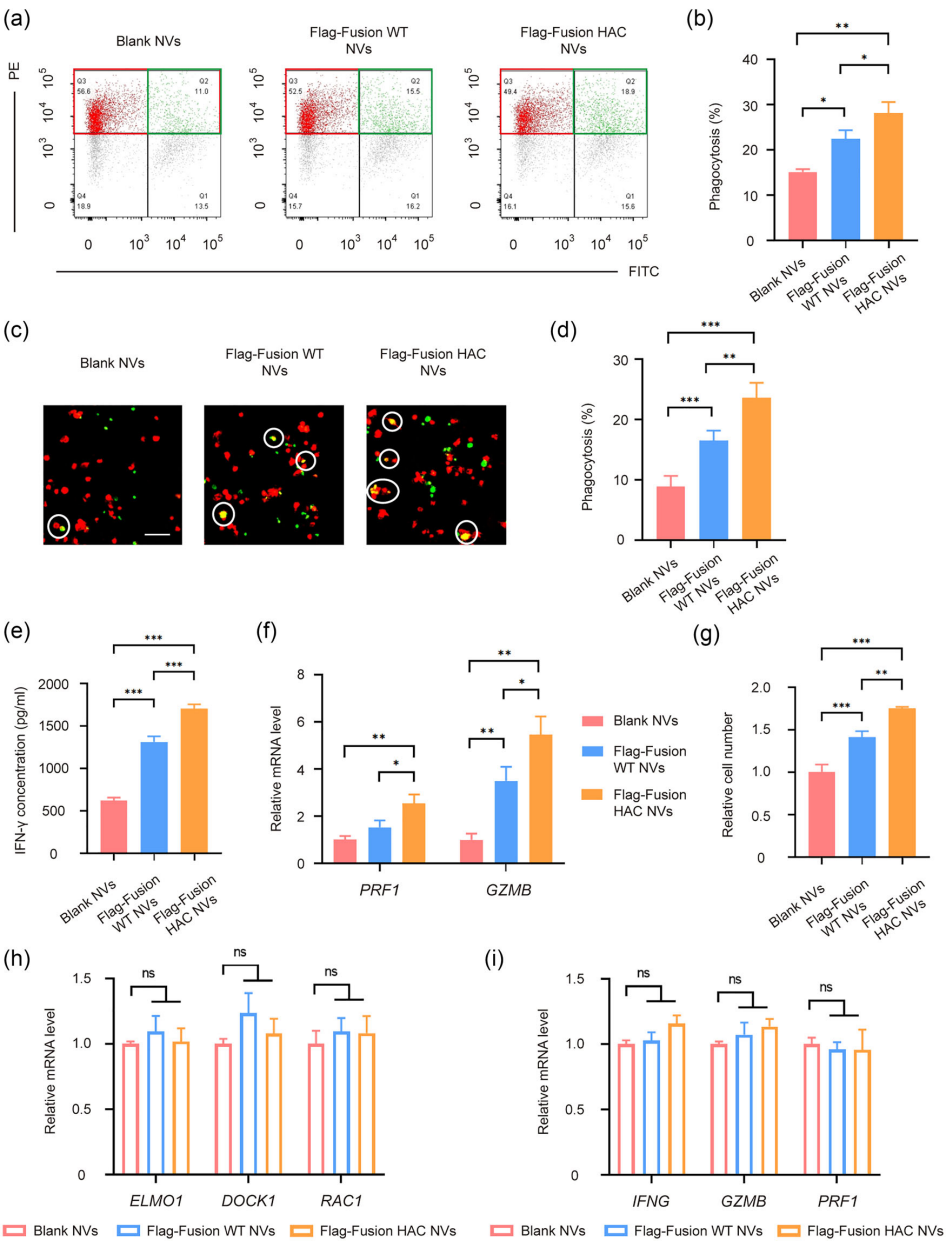
HAC NVs block CD47 and PD‐L1 checkpoints to promote antitumor immunity in vitro. (A–D) MDA‐MB‐231 cells were treated with 100 μg Blank, Flag‐Fusion WT or Flag‐Fusion HAC NVs for 12 h, then co‐cultured with THP‐1 derived macrophages for 24 h in serum‐free medium. (a) Representative plots showing phagocytosis assays analysed by flow cytometry. Phagocytosis was quantified as the percentage of macrophages (red gate) that became FITC^+^ (green gate). (b) Quantitative analysis of phagocytosis of tumour cells by macrophages was assessed by flow cytometry in (a) (*n* = 3, *p < 0.05, **p < 0.01). (c) Representative fluorescence images of phagocytosis assays. White circles indicate MDA‐MB‐231 cells (Green) swallowed by macrophages (Red). Scale bar: 100 μm. (d) Quantitative analysis of the phagocytosis of MDA‐MB‐231 cells by THP‐1 derived macrophages in (c) (*n* = 5, **p < 0.01, ***p < 0.001). (e) MDA‐MB‐231 cells were first incubated with Blank, Flag‐fusion WT or Flag‐fusion HAC NVs at the concentration of 100 μg and then co‐cultured with human CD8^+^ T cells at the ratio of 1:1 for 48 h. The concentration of IFN‐γ in the culture supernatants were measured by ELISA (*n* = 5, ***p < 0.001). (f) qPCR analysis of granzymes B and perforin production by cytotoxic T cells in each group (*n* = 3, *p < 0.05, **p < 0.01). (g) Cell viability of T cells for 48 h after co‐cultured with MDA‐MB‐231 in each group (*n* = 5, **p < 0.01, ***p < 0.001). (h) THP‐1 derived macrophages were treated with 100 μg Blank, Flag‐Fusion WT or Flag‐Fusion HAC NVs for 24 h. Statistics analysis of the mRNA expression of cytotoxicity related proteins in T cells directly incubated with NVs were examined by RT‐qPCR (*n* = 3). (i) CD8^+^ T cells were treated with 100 μg Blank, Flag‐Fusion WT or Flag‐Fusion HAC NVs for 24 h. Statistics analysis of the mRNA expression of phagocytosis related proteins in macrophages directly incubated with NVs were examined by RT‐qPCR (*n* = 3).

Apart from immune‐independent tumour suppression, we further verified the effect of innate and adaptive anti‐tumour immunity through blocking the SIRPα/CD47 and PD‐1/PD‐L1 pathway in vitro. To test whether fusion HAC NVs could trigger the macrophage phagocytosis of tutor cells, MDA‐MB‐231 cells were treated with 100 μg blank NVs, fusion WT NVs or fusion HAC NVs, and then co‐cultured with THP‐1 derived macrophages. Flow cytometry revealed that the phagocytosis ability of macrophages after the fusion HAC NVs treatment was improved ∼1.8‐fold and ∼1.2‐fold, compared with that in blank NVs and fusion WT NVs groups (Figure 5a,b). Similar increase trend also visually detected by confocal laser scanning microscope (Figure 5c,d). To exclude the increased phagocytosis of macrophages is the result of the removal of apoptotic tumour cells, we detected apoptosis in MDA‐MB‐231 tumour cells incubated with blank, fusion WT, or fusion HAC nanovesicles (NVs) for 12 h. We found that there was no significant apoptosis occurred in tumour cells in each group after 12 h NVs treatment (Figure S7A,B). Next, we detected CD47‐SIRPα‐independent phagocytosis signalling pathways in macrophages mentioned above, and found no significant differences in protein expression of ELMO‐DOCK1‐Rac1 pathway between the three groups (Figure S7C). These results revealed that macrophage clearance of tumours caused by HAC NVs were the inhibition of CD47‐SIRPα immune checkpoints, rather than phagocytosis of apoptotic cells.

Moreover, to detect whether fusion HAC NVs could activate T‐cell immunity, MDA‐MB‐231 cells were treated with blank NVs, fusion WT NVs or fusion HAC NVs, and then co‐cultured with peripheral blood mononuclear cell (PBMC) derived T cells. The concentration of IFN‐γ in the culture supernatants were measured by enzyme linked immunosorbent assay (ELISA), which was about 619.6, 1313 and 1706 pg/mL after treatment with blank NVs, fusion WT NVs and fusion HAC NVs, respectively. A significant increase of IFN‐γ was measured after the fusion HAC NVs treatment, indicating potent T‐cell activation (Figure 5e). In addition, the expression levels of perforin and granzyme B in fusion HAC NVs treated T cells were ∼2.6‐fold and ∼5.5‐fold higher, while fusion WT NVs were only ∼1.5‐fold and ∼3.5‐fold higher than blank NVs group (Figure 5f). Furthermore, the viability of T cells treated with fusion HAC NVs was increased by cell counting in comparison with that in blank NVs and fusion WT NVs groups (Figure 5g). These results demonstrated that the fusion HAC NVs can block CD47 and PD‐L1 checkpoints to promote phagocytosis of macrophages and tumour‐killing effect of T‐cells to trigger robust antitumour immunity in vitro.

To investigate whether fusion WT NVs and fusion HAC NVs have any direct effect on immune cells, we treated macrophages and T cells with blank, fusion WT or fusion HAC NVs, and found that there was no significant change in the expression of immune cell function related proteins in the fusion WT NVs and fusion HAC NVs groups compared with that in control group (Figure 5h,i). These results further confirmed that the inhibitory effect of fusion WT and fusion HAC NVs on tumour growth was due to blocking the immune checkpoint protein interaction between tumour cells and immune cells.

### 3.5 HAC NVs inhibit immune‐independent tumour proliferation in vivo

**FIGURE 6:**
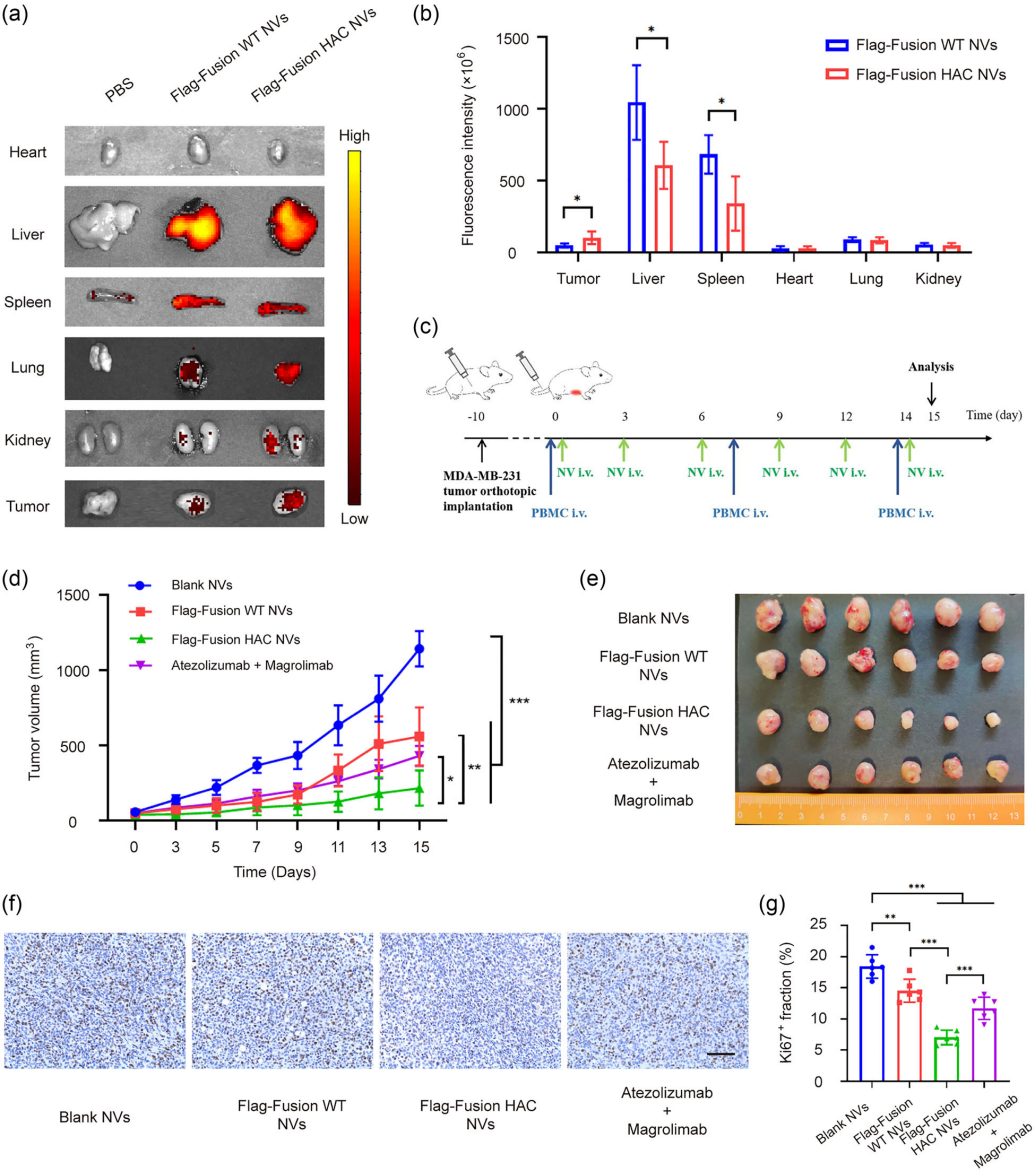
Dual blockade of CD47 and PD‐L1 with HAC NVs inhibits tumour proliferation in vivo. (a) Representative ex vivo fluorescent images of the DiR‐labelled Blank, Flag‐Fusion WT or Flag‐Fusion HAC NVs in tumours and main organs. (b) Statistical analysis of fluorescence intensity of different organs and tumours in (a) (*n* = 5, *p < 0.05). (c) Schematic schedule of therapeutic study in MDA‐MB‐231 xenograft mouse model. (d) Tumour growth curves of MDA‐MB‐231 tumour‐bearing mice with treatment of NVs or antibodies (*n* = 6, *p < 0.05, **p < 0.01 and ***p < 0.001). (e) Photograph of tumour tissues obtained from different groups (*n* = 6). F. Ki‐67 levels in tumour tissues of each group presented in representative immunohistochemistry images. Scale bar: 50 μm. (g) Quantification of Ki‐67 levels in (f) (*n* = 6, **p < 0.01, and ***p < 0.001).

After confirming the abilities of fusion HAC NVs in immune modulation, the antitumour effect of these NVs in vivo was further investigated. An important feature of NVs is their ability to be naturally recruited by tissues and involved in physiological processes (Karasu et al., 2018). We cultured MDA‐MB‐231 multicellular spheres with an appropriate size, and then incubated with DiO‐labelled NVs. Confocal imaging showed that the penetration of fusion HAC NVs to tumour spheres was significantly increased, compared with fusion WT NVs (Figure S8). In order to detect the in vivo biodistribution of fusion WT NVs and fusion HAC NVs, DiR‐labelled NVs were injected into the tumour‐bearing mice via the tail vein. As compared with fusion WT NVs, fusion HAC NVs exhibited reduced accumulation in liver and spleen, and enhanced accumulation in tumour (Figure 6a,b). These results confirmed that fusion HAC NVs could effectively penetrate into tumour tissues, thereby playing an important antitumour role.

Biological activity of fusion WT NVs and fusion HAC NVs were evaluated using co‐implantation mice model. Specifically, mice were implanted with MDA‐MB‐231 cells and injected human PBMC once a week, followed by administration of fusion WT NVs, fusion HAC NVs, or controls. Intravenous injection (i.v.) of 10 mg/kg fusion WT NVs or fusion HAC NVs were performed three times a week. In control groups, 10 mg/kg blank control NVs or 1 mg/kg Atezolizumab and 1 mg/kg Magrolimab combination (mAbs) were administrated i.v. (Figure 6c). We found that fusion WT NVs, fusion HAC NVs and mAbs could all inhibited tumour growth (Figure S9A). Among the three groups, the inhibitory effect of fusion HAC NVs was the most significant, the tumour mean volume was reduced from 559.7 to 430.2 mm^3^ treatment with fusion WT NVs and mAbs to 216.1 mm^3^ with fusion HAC NVs (Figure 6d,e). The efficacy of different treatments was also evaluated by the tumour weight, which showed similar change trend that fusion HAC NVs treatment reached better efficacy than other treatments (Figure S9B).

In addition, the least ratio of Ki67 positive cells and the highest TUNEL fluorescence intensity suggested that fusion HAC NVs could reduce the proliferation (Figure 6f,g) and promote apoptosis (Figure S10A,B) in tumour microenvironment (TME), which could further prove the remarkable treatment effects of fusion HAC NVs. These results illustrated that fusion HAC NVs could significantly inhibit tumour growth through blocking PD‐L1 and CD47 signalling simultaneously in vivo.

### 3.6 Dual blockade of CD47 and PD‐L1 with HAC NVs elicits potent antitumour immunity

**FIGURE 7:**
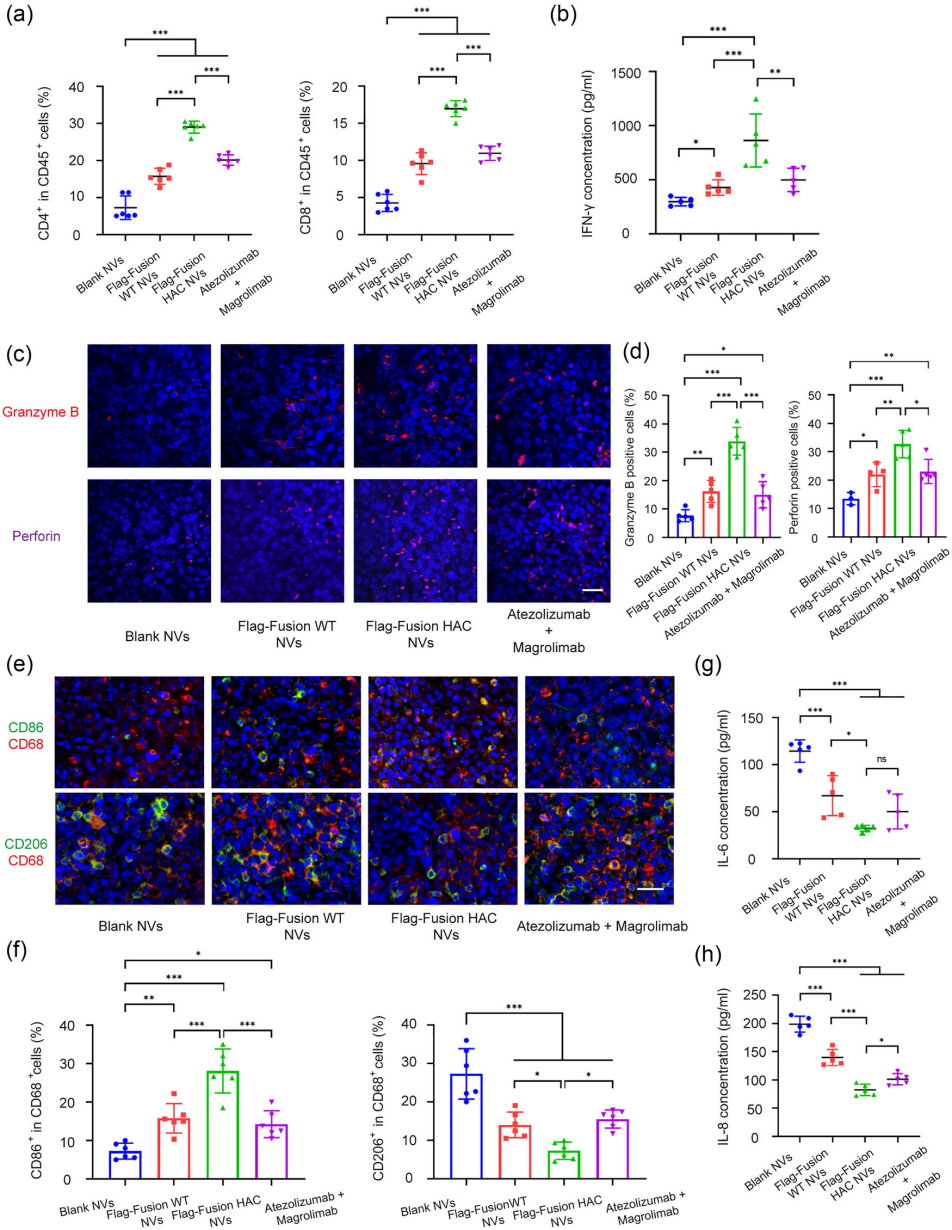
Dual blockade of CD47 and PD‐L1 with HAC NVs elicits potent antitumour immunity. (a) Flow cytometric quantification of CD4^+^ and CD8^+^ T cells in tumour from each group after indicated administration (*n* = 6, ***p < 0.001). (b) ELISA measurement of IFN‐γ in plasma from each group after indicated administration (*n* = 5, *p < 0.05, **p < 0.01 and ***p < 0.001). (c) Representative immunofluorescence images of granzyme B and perforin in tumour tissues after indicated administration (Red: granzyme B; Magenta: perforin; Blue: DAPI). Scale bar: 50 μm. (d) Statistical analysis of granzyme B and perforin in tumour samples in (c) (*n* = 3–5, *p < 0.05, **p < 0.01 and ***p < 0.001). (e) Representative immunofluorescence images of CD86^+^ M1 and CD206^+^ M2 macrophages in tumour tissues after indicated administration (Red: CD68; Green: CD86, CD206; Blue: DAPI). Scale bar: 50 μm. (f) Statistical analysis of M1 and M2 macrophages in tumour samples in (e) (*n* = 6, *p < 0.05, ***p < 0.001). (g and h) ELISA measurement of IL‐6 (g) and IL‐8 (h) in plasma from each group after indicated administration (*n* = 5, *p < 0.05, ***p < 0.001).

In order to study the immune system in mice model, we collected the peripheral blood of mice and detected the proportion of human derived CD45^+^ immune cells. Compared with the control group injected with saline, we found that more than 20% of human derived immune cells were present in mice peripheral blood after 3 weeks of PBMC injection in the fusion HAC NVs group as the experimental group. This indicated that the human immune reconstitution in the mice model was successful (Figure S11A,B). To further explore the changes in TME after in vivo fusion HAC NVs treatment, we isolated the tumour tissues and extracted the infiltrating immune cells. Firstly, we performed flow cytometry to analyse the changes of T cell‐mediated adaptive anti‐tumour immunity. We found that the proportion of CD4^+^ T cells in the fusion HAC NVs group was significantly increased compared with that in other groups, which was ∼1.8‐fold and ∼1.5‐fold of that in fusion WT NVs and mAbs combination group, respectively. And the proportion of CD8^+^ T cells showed a similar trend (Figure S12 and Figure 7a). Additionally, we performed immunofluorescence staining of CD4 and CD8 in tumour sections, and found that the proportion of CD4^+^ or CD8^+^ T cells in the fusion HAC NVs group were significantly increased compared with that in fusion WT NVs and mAbs combination groups, which was consistent with the flow cytometry results (Figure S13A,B). Meanwhile, ELISA detected the plasma levels of IFN‐γ produced by CD8^+^ T cells in fusion HAC NVs group was 863 pg/mL and significantly higher than that in other groups, which was 298, 430 and 500 pg/mL, respectively (Figure 7b). Next, we performed immunofluorescence staining of granzyme B and perforin, which indicate T‐cell killing activity. We found that the ratio of granzyme B and perforin positive cells were significantly increased than other groups, which were ∼2‐fold and ∼1.4‐fold of the fusion WT NVs and mAbs group (Figure 7c,d).

Additionally, as for innate immunity, tumour associated‐macrophages (TAMs) can be divided into two main subtypes namely M1 anti‐tumour macrophages and M2 pro‐tumour macrophages (Zhou et al., 2020). Immunofluorescence staining revealed a significant reduction on ∼4.5‐fold in CD206^+^ M2 phenotype macrophages and an increase on ∼4‐fold in CD86^+^ M1 phenotype after fusion HAC NVs treatment compared with blank NVs, suggesting a down‐regulation of tumour immunosuppression (Figure 7e,f). Moreover, plasma levels of the pro‐tumour cytokines IL‐6 and IL‐8 produced by macrophages in fusion HAC NVs group were the lowest in all groups, which were reduced ∼72% and ∼59% in comparison with blank NVs (Figure 7g,h). The gene expression results in tumour tissues were consistent with the above results (Figure S14). These results demonstrated that fusion HAC NVs could reprogram tumour‐associated macrophages from pro‐tumour (M2) to anti‐tumour (M1) type, and promote the tumour‐killing effect of cytotoxic T lymphocyte, thus improving the immunosuppressive state in TEM from both innate immunity and adaptive immunity aspects.

Furthermore, the mice received i.v. injections of NVs and mAbs were studied for in vivo toxicity. Neither death nor an obvious body weight difference was observed between the different groups over 15 days, demonstrating that no detectable side effects were induced by fusion HAC NVs (Figure S15). Plasma biochemistry and blood routine test were carried out on day 15 post the fusion HAC NVs treatment (Figure S16 and Table S2). Together with histological examination of major organs harvested on day 15 post the treatment, we demonstrated that i.v. injection of fusion HAC NVs did not induce drastic side effects to experimental animals (Figure S17). Therefore, fusion HAC NVs could be a safety and promising antibodies delivery system for blocking CD47 and PD‐L1 checkpoints and eliciting robust innate and adaptive immunity.

## 4 DISCUSSION

With the exhilarating efficacy of immunotherapy in melanoma, lung cancer, breast cancer and other malignant tumours, the ICB therapy against PD‐1 or PD‐L1 has become a clinical hot spot (Garon et al., 2015; Patnaik et al., 2015; Topalian et al., 2014). However, only minority (10%–35%) of cancer patients can benefit from mono‐targeting therapy, since there are many immune check points beyond PD‐1/PD‐L1 axis. Thus, exploring potential targets to partner PD‐1/PD‐L1 antibody becomes a better choice. As an innate immune checkpoint, CD47 on tumour cells can interact with the signal regulatory protein SIRPα on the surface of macrophages and send a ‘don't eat me’ signal to assist tumour cells to escape immune surveillance. Therefore, SIRPα/CD47 axis has naturally become an attractive target (Hatherley et al., 2008).

Interestingly, both PD‐L1 and CD47 are highly expressed in tumour cells and can be synchronously regulated by transcription factor MYC (Casey et al., 2016). Recent studies have demonstrated that targeted blockade of both CD47 and PD‐L1 on tumour cells with a bispecific anti‐PD‐L1‐CD47 (SIRPα) showed significantly enhanced tumour targeting and therapeutic efficacy versus monotherapy (Chen, Dominik, et al., 2021; Liu, Guo, et al., 2018; Liu, Liu, et al., 2018). However, most antibodies have limited ability to penetrate and accumulate in tumour tissues due to their large size, making it difficult to maintain effective therapeutic concentrations in target tissues (Thakur et al., 2018). Previous researches suggested that nanovesicles (NVs) overexpressing high‐affinity PD‐1 or SIRPα variants, which could push the limit of current therapeutics, significantly alleviated the immunosuppressive state of tumour and delayed tumour growth (Chen et al., 2022; Rao et al., 2020; Zhang et al., 2018). There have been no studies to integrate SIRPα and PD‐1 high‐affinity consensuses (HAC) on the same NVs. Therefore, in this study, we engineered dual‐targeted cellular HAC NVs instead of fusion proteins and antibodies to circumvent their poor tissue/tumour penetrance and complicated isolation technology, and compared the immunotherapy efficacy of them with fusion WT NVs and monoclonal antibodies on tumours in vitro and in vivo. Our fusion HAC NVs could effectively block both PD‐L1 and CD47 checkpoints on tumour cells to elicit antitumour immunity of T cell and macrophage respectively (Figures 3 and 5). Moreover, in animal study, the ICB therapeutic efficacy of fusion HAC NVs was superior to the combination of Atezolizumab (anti‐PD‐L1) and Magrolimab (anti‐CD47) (Figures 6 and 7).

To improve ICB blocking efficiency, recent studies have used directed evolution by yeast‐surface display to select the ectodomain of PD‐1 or SIRPα as a high‐affinity competitive antagonist of its ligand. The beneficial mutated clones showed a 400–500‐fold increase in affinity for hPD‐L1 and about a 50000‐fold increase in affinity for hCD47 compared with the corresponding wild‐type consensus as measured by surface plasmon resonance (Maute et al., 2015; Weiskopf et al., 2013). With these high affinity consensuses, we designed the fusion HAC NVs and compared them with fusion WT NVs expressing wild‐type PD‐1 and SIRPα. Our data revealed that fusion HAC NVs had significantly higher affinity for PD‐L1 and CD47 on tumour cells than fusion WT NVs, which promoted the antitumour function of macrophages and T cells both in vitro and in vivo. Encouragingly, fusion HAC NVs have better efficacy than antibody combination in animal experiments. Currently, although antibodies engineered to have higher affinity have not been commercialised and are difficult to compare with our fusion HAC NVs, we speculated that the affinity of fusion HAC NVs to tumour cells might be consistent with HAC antibodies according to fusion WT NVs results. However, compared with antibodies and fusion proteins, the advantage of fusion HAC NVs was that they could be further used to construct drug delivery systems and further enhance antitumour therapies (Yong et al., 2022).

Till now, several cell types were used to produce NVs, including tumour cells, erythrocytes and stem cells (Meng et al., 2021; Zhang et al., 2021). The characteristic of tumour cells was that the NVs they produced could inherit the homology of the source cells and were more likely to target homologous tumour cells. This led to differences in the downstream effects of tumour cell derived NVs for different types of tumours (Rao et al., 2020). Therefore, we used HEK293 cells (human embryonic kidney cells) to produce NVs in our study, which were easy to expand in vitro, perform engineering and could function on different types of tumour cells with high biological safety (Zhang et al., 2018). Additionally, there are significant differences in the expression levels of PD‐L1 and CD47 on tumour cells with different malignancy and NVs internalised into cells after anchoring to ligands on the surface of target high malignance cells by high‐affinity PD‐1 and SIRPα proteins, suggesting that these fusion HAC NVs were potential for targeted drug delivery application (Bian et al., 2022; Li et al., 2023; Zhang et al., 2018).

In addition to immune suppression, PD‐L1 plays not only a role in the interaction with PD‐1 on lymphocytes, but also as an important molecule involved in tumour proliferation, which is considered as a marker indicating tumour aggressiveness (Stefan Kraft et al., 2017). Recent reports showed that Atezolizumab exerted inhibitory effects on TNBC, thereby inhibiting EMT/metastasis and tumour growth (Chen, Li, et al., 2021; Saleh et al., 2019). The similar biological effects on cancer cells were also found by blocking PD‐L1 with high affinity PD‐1 variant (havPD‐1) overexpressing EVs (Chen et al., 2022). Furthermore, CD47 inhibition could also inhibit proliferation and induce apoptosis of tumour cells (Hu et al., 2020; Zhang et al., 2020). Similar inhibition effects on tumour cells were observed in our fusion HAC NVs, which may also affect intracellular signalling of PD‐L1 and CD47. The fusion HAC NVs inhibited tumour cells proliferation and migration and promoted tumour cells apoptosis. And their effect was more significant than those of fusion WT NVs as a control (Figure 4).

The NVs engineered from cell line obtain tumour‐ or tissue‐specific accumulation, easy translocation through physical barriers and extracellular matrix, excellent biosafety and stability (Andaloussi et al., 2013; Rao et al., 2020). Our fusion HAC NVs were derived from the plasma membrane of HEK293 cells with more than 95% NVs expressing well‐distributed exogenous SIRPα‐PD‐1 HAC protein, which is higher than that resulted from fusion of membrane with single component (Meng et al., 2021). In this study, there were no significant difference in protein marker was found between naturally produced EVs and NVs formulated by sonication and extrusion, which was in line with reported data (Wang et al., 2019; Wen et al., 2022). However, the content of fusion WT or fusion HAC proteins in NVs was much higher than that in EVs. The reason for this difference may be that compared with the complex membrane composition of EVs, the membrane source of NVs was mainly the plasma membrane with high expression of PD‐1 and SIRPα fusion proteins (Gandham et al., 2020; Shao et al., 2018). In addition, NVs had the obvious advantage ∼200‐fold higher yield and ∼100‐fold faster production than those of collection of naturally secreted EVs and could guarantee subsequent clinical application (Molinaro et al., 2016; Jang et al., 2013). Another attractive feature of these fusion WT NVs is that protein components can be severally customised, providing high freedom in programmable construction of fusion proteins. It is conceivable that the fusion WT NVs platform can be extended to concurrently targeting to other checkpoints for synergetic cancer immunotherapy except PD‐1/PD‐L1 and SIRPα/CD47, such as CTLA‐4, LAG‐3, TIGIT and so on (Kubli et al., 2021; Rudd et al., 2009; Yu et al., 2009). Furthermore, one of the important functions of NVs in clinical application is drug package and delivery. NVs can be applied in immunotherapy concurrent with chemotherapy, including gemcitabine, cisplatin and paclitaxel (Jang et al., 2013; Wan et al., 2018). The therapeutic NVs we developed deserve further investigation in drug delivery, which will expand the portfolio of them as immunotherapeutic agents.

## AUTHOR CONTRIBUTIONS

**Luyao Zhang**: Data curation; formal analysis; methodology; investigation; writing—original draft; writing—review and editing. **Xu Zhao**: Formal analysis; methodology; investigation; software; writing—original draft; writing—review and editing. **Yanan Niu and Xiaoya Ma**: Investigation; validation. **Wei Yuan**: Formal analysis; writing—review and editing; supervision; funding acquisition. **Jie Ma**: Formal analysis; writing—review and editing; supervision; funding acquisition. All authors have agreed to publish the manuscript.

## CONFLICT OF INTEREST STATEMENT

The authors declare that they have no competing interests.

## Supporting information

Supporting Information

Supporting Information

Supporting Information

## Data Availability

The data that support the findings of this study are available from the corresponding author upon reasonable request.
