# Supplementary material for: Engineering high‐affinity dual targeting cellular nanovesicles for optimised cancer immunotherapy
Source: J Extracell Vesicles. 2023 Nov 16;12(11):12379. doi: 10.1002/jev2.12379 (PMC10654473; doi:10.1002/jev2.12379)
Supplement: Supplementary file 1 — Supporting Information [file JEV2-12-12379-s001.docx]

**Supporting Information**

# Engineering high-affinity dual targeting cellular nanovesicles for optimized cancer immunotherapy

Luyao Zhang ^1, †^, Xu Zhao ^2, †^, Yanan Niu ^2^, Xiaoya Ma ^2^, Wei Yuan ^2, *^, Jie Ma ^1, *^

*^1^ Center of Biotherapy, Beijing Hospital, National Center of Gerontology, Institute of Geriatric Medicine Chinese Academy of Medical Sciences, Beijing, 100730, P.R. China.*

*^2^ State Key Laboratory of Molecular Oncology, National Cancer Center/National Clinical Research Center for Cancer/Cancer Hospital, Chinese Academy of Medical Sciences and Peking Union Medical College, Beijing, 100021, P.R. China.*

*^†^ These authors contributed equally to this work.*

*^*^ Corresponding authors.*

*E-mail:* [*majie4685@bjhmoh.cn*](mailto:majie4685@bjhmoh.cn)*, majie@cicams.ac.cn (Jie Ma); yuanwei@cicams.ac.cn (Wei Yuan)*

**This file includes:**

Materials and Methods

Figs. S1 to S17

References

**Materials and methods**

**Isolation of extracellular vesicles (EVs)**

We transfected HEK293 cells with an expression plasmid to construct engineered cells. To collect EVs, cells were washed with PBS and incubated for 24 h in DMEM-high glucose medium supplemented with 10% exosome-free fetal bovine serum (System Biosciences, USA). The medium was then collected, and EVs were isolated by standard differential centrifugation protocol (Théry et al., 2006; Wen et al., 2020). Briefly, medium was successively centrifuged at 500 g for 10 min, 2,000 g for 10 min and 10,000 g for 30 min to remove floating cells, cellular debris and large vesicles, respectively. The supernatant was then ultracentrifuged at 100,000 g for 70 min. EVs pellets were washed with PBS, centrifuged again at 100,000 g for additional 70 min.

**Nanovesicles cell binding and uptake assay**

To evaluate the binding and uptake capacity of HAC NVs in different cell lines, ~5 × 10^4^ HEK 293, MCF-7 and MDA-MB-231 stained with Hoechst 33342 were seeded in a 24-well plate, respectively. After incubation for 24 h, different cells were treated with the same amount of green fluorescent labeled HAC NVs for 6 h. After that, free nanovesicles were washed and removed carefully with PBS three times. Using fluorescent microscope to investigate the binding and uptake of HAC NVs in different cell lines.

**Immune reconstitution efficiency detection**

For detection of immune reconstitution efficiency in mice model, whole blood was collected in control group (saline injection) and experimental group (PBMC injection) for staining with PC5.5-anti-Human-CD45 antibody for 20 min. The samples were then lysed with red blood cell lysis buffer (BD Pharmingen, USA) at room temperature for 10 min. The proportion of human immune cells in peripheral blood of mice was detected by Flow Cytometry after centrifugation.


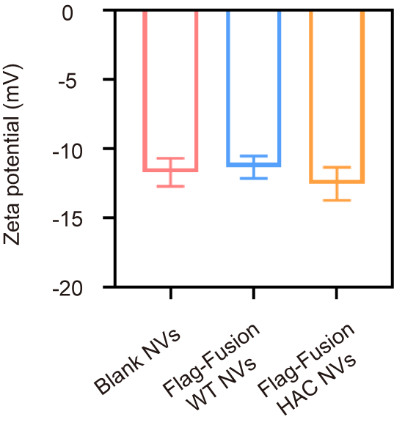


**Figure S1.** The surface ζ potential of Blank, Flag-Fusion WT, and Flag-Fusion HAC NVs.


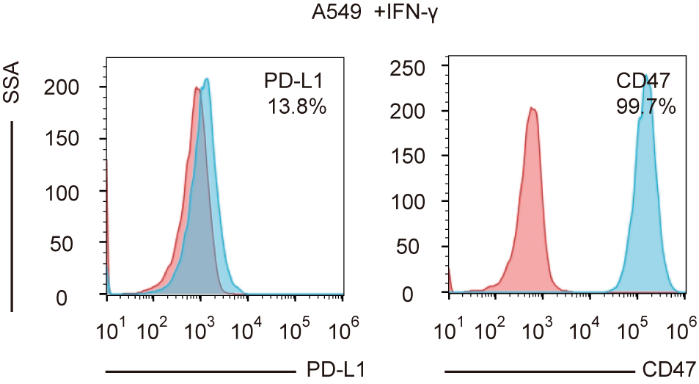


**Figure S2.** The expression level of PD-L1 and CD47 was measured by flow cytometry in A549 (Red: Isotype antibody; Blue: anti-PD-L1, or anti-CD47 antibody).


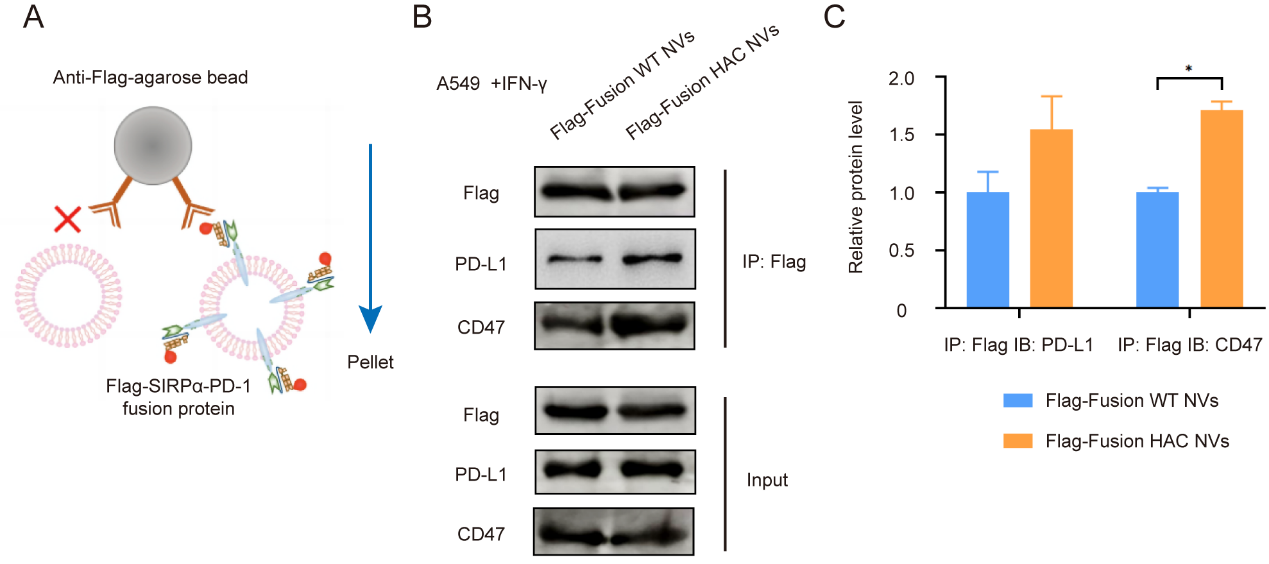


**Figure S3.** **A)** Schematic illustration for Flag antibody to pull down the Flag-SIRPα-PD-1 NVs. **B)** Representative western blot analysis of co-immunoprecipitation of Blank, Flag-Fusion WT, or Flag-Fusion HAC NVs interacting with PD-L1 and CD47 in A549. **C)** Statistics analysis of immunoprecipitation in (B) (n=2, **P*<0.05).


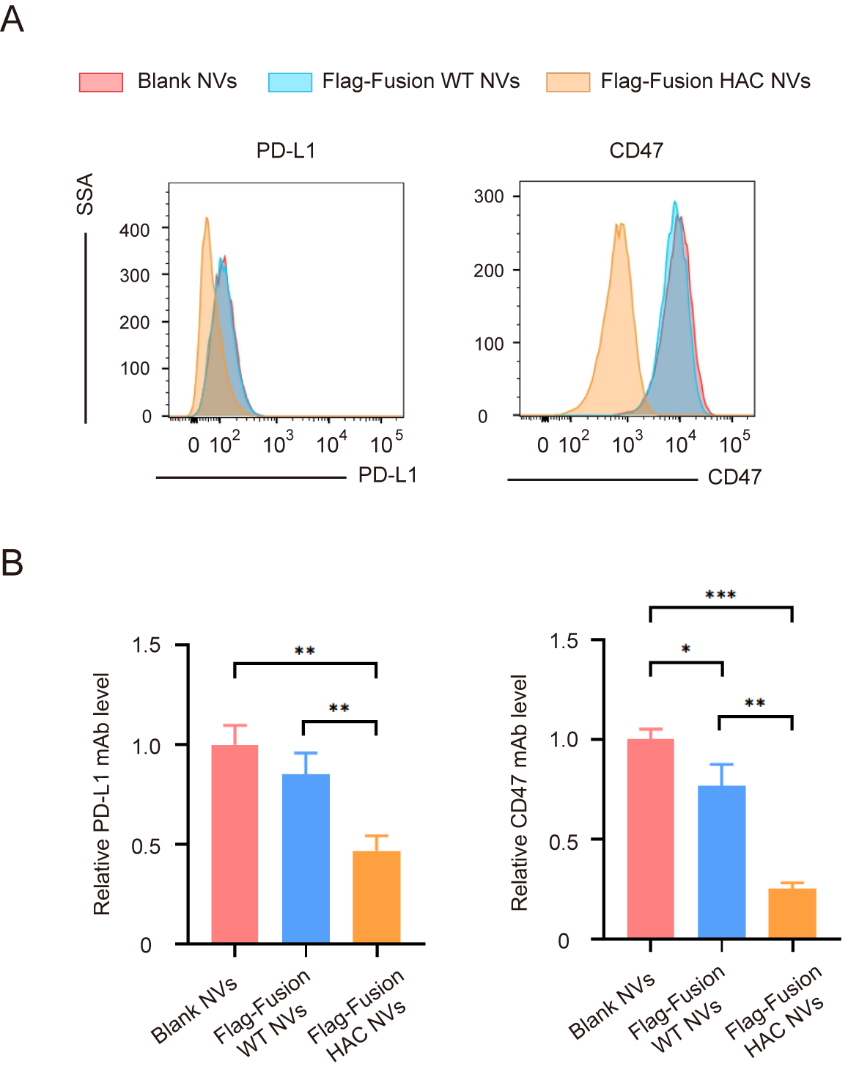


**Figure S4.** **A)** Binding and blocking efficiency of Blank, Flag-Fusion WT, or Flag-Fusion HAC NVs to PD-L1 and CD47 was measured by flow cytometry in A549. **B)** Quantitative analysis of PD-L1 or CD47 competing binding efficiency against NVs (A) (n=3, **P*<0.05, ***P*< 0.01, and ****P*< 0.001).


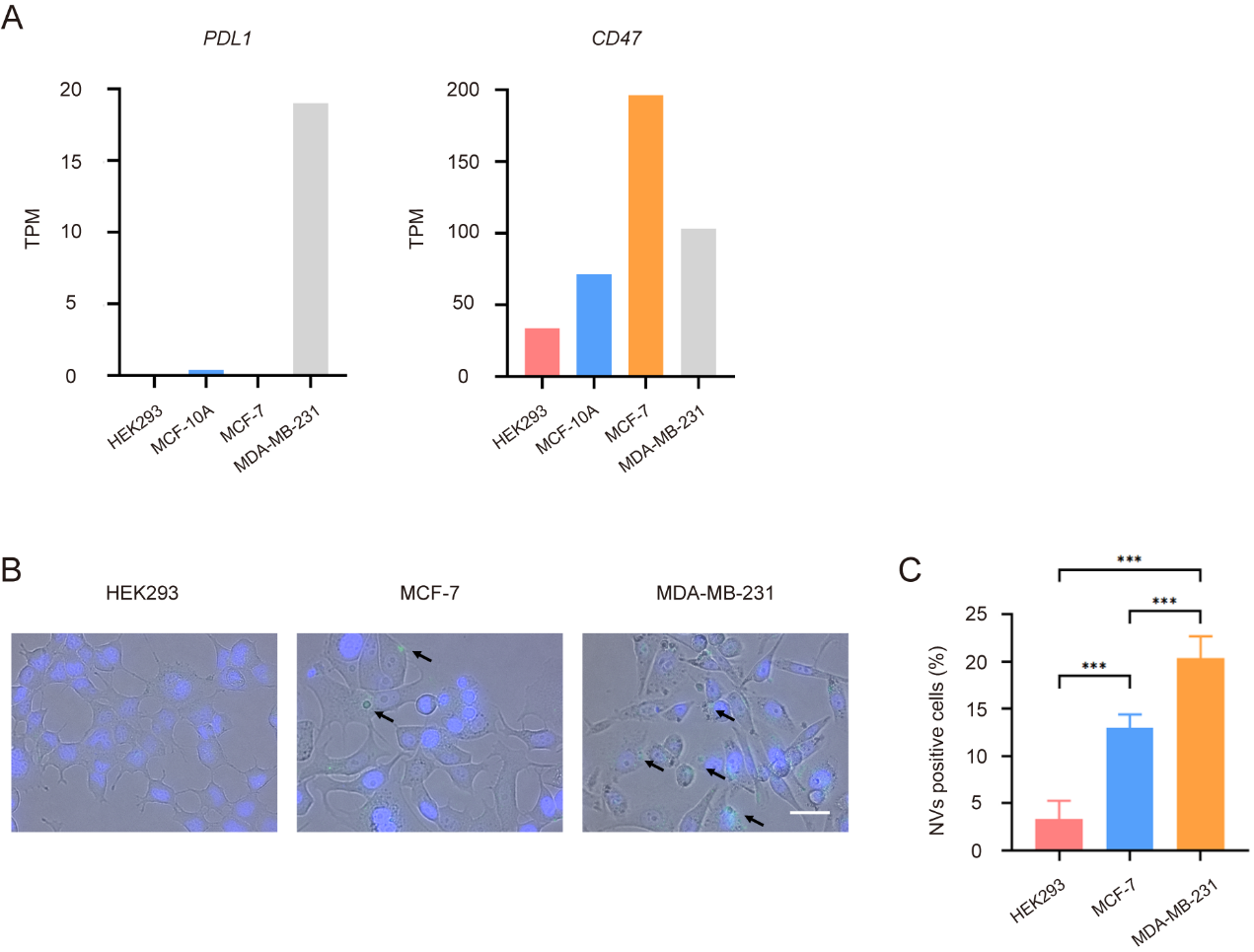


**Figure S5.** **A)** *PDL1* and *CD47* expression in different cell lines in Human Protein Atlas (HPA) database. **B)** Fluorescence microscopy images of binding and uptake ability of Flag-Fusion HAC NVs by different cell lines for 6 h. Scale bar: 50 µm. **C)** Statistics analysis of NVs binding and uptake (B) (n=5, ****P*< 0.001).


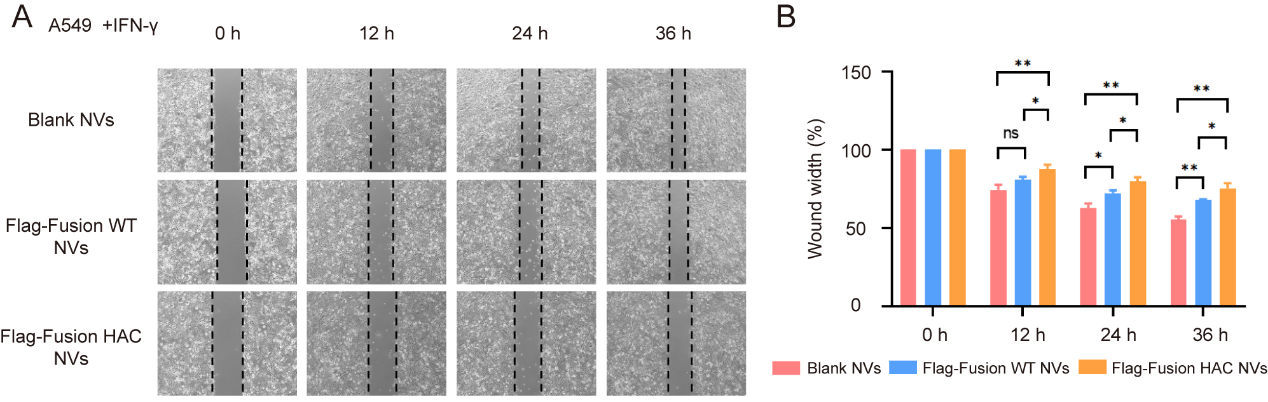


**Figure S6.** **A)** Wound healing assay of A549 cells treated with Blank, Flag-Fusion WT, or Flag-Fusion HAC NVs, respectively. Migration was evaluated at 0, 12, 24 and 36 h time points after wounding, respectively. **B)** Statistics analysis of wound closure in (A) (n=3, **P*<0.05, ***P*< 0.01).


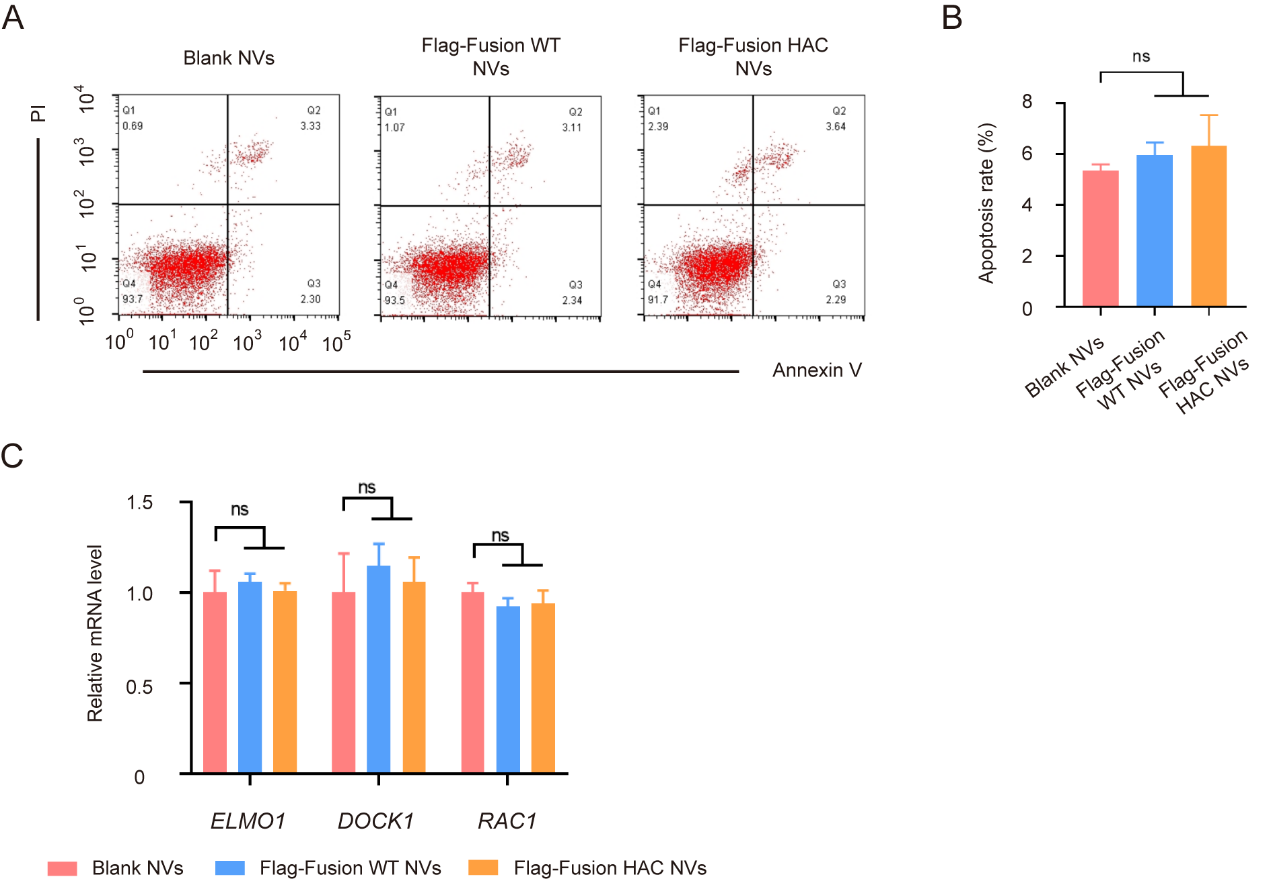


**Figure S7.** **A)** Apoptotic status of MDA-MB-231 cells treated with 100 μg Blank, Flag-Fusion WT, or Flag-Fusion HAC NVs for 12 h. **B)** Statistics analysis of cells apoptosis in (A) (n=3). **C)** Statistics analysis of the mRNA expression of phagocytosis related proteins in macrophages incubated with MDA-MB-231 treated with NVs for 12 h were examined by RT-qPCR (n=3).


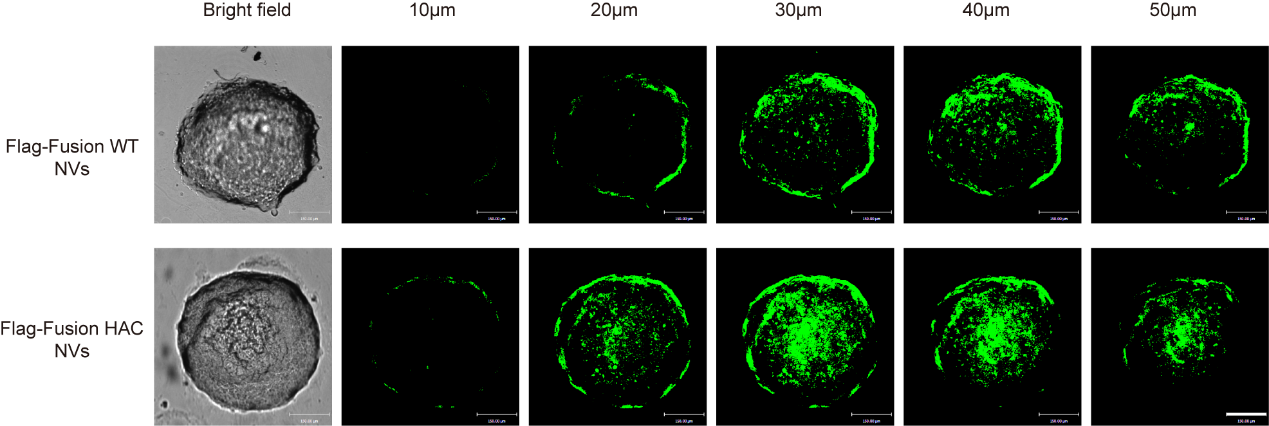


**Figure S8.** Penetration level of Flag-Fusion WT, and Flag-Fusion HAC NVs in multicellular MDA-MB-231 tumor spheroids (Green: DiO-labeled NVs). Scale bar: 150 μm.


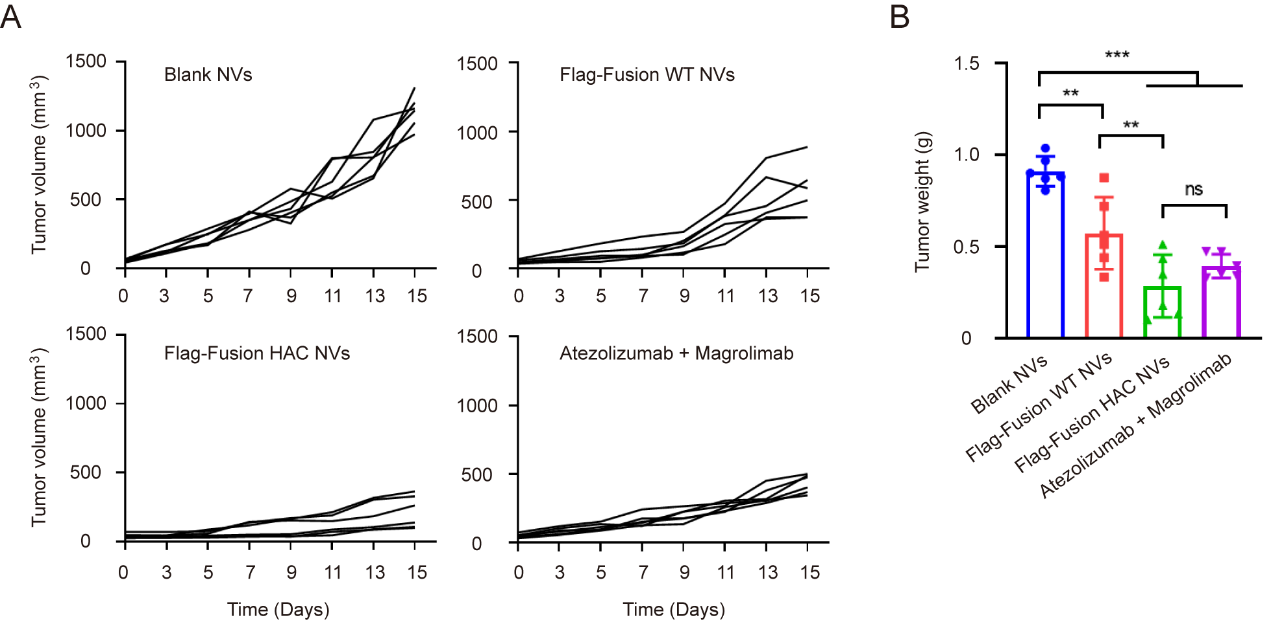


**Figure S9.** **A)** Change curve of individual tumor growth and **B)** Tumor weight treated with different treatments (n=6, ***P*< 0.01, ****P*< 0.001).


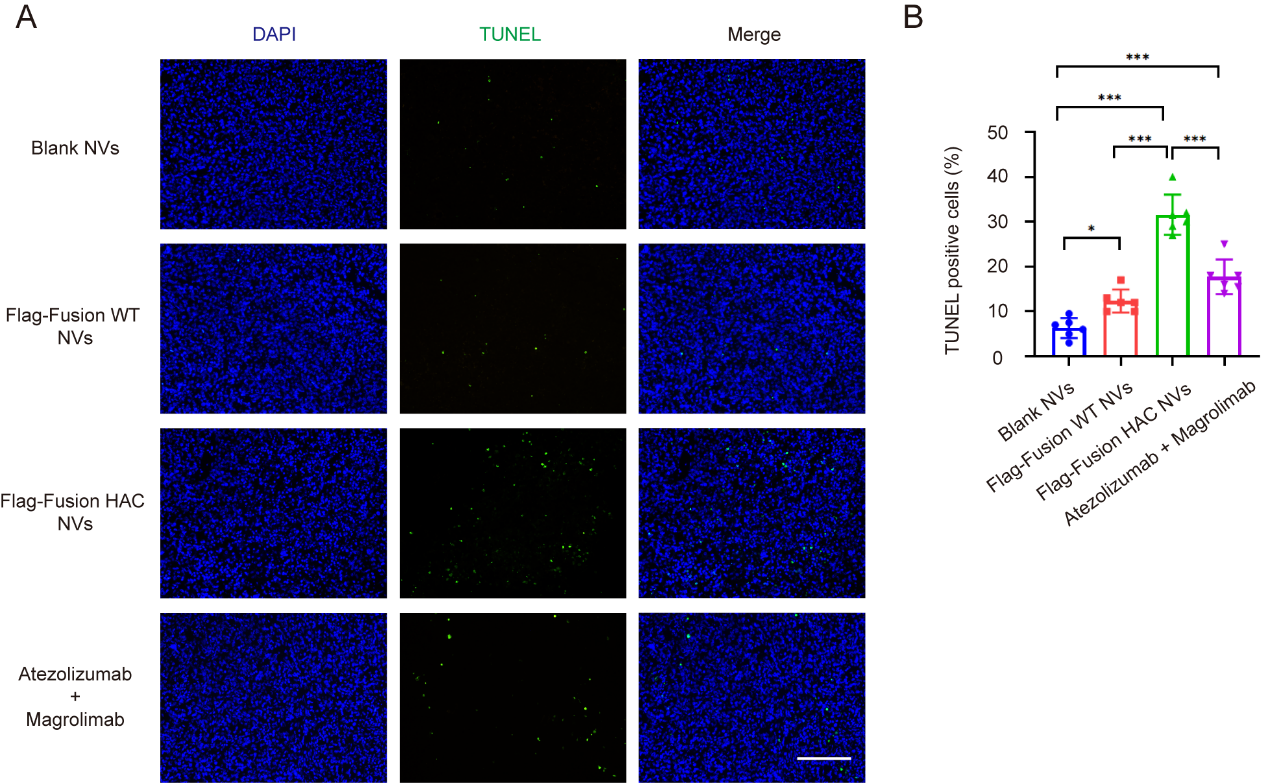


**Figure S10.** **A)** The fluorescent images of TUNEL staining of tumor tissues and **B)** Statistics analysis of TUNEL positive cells after different treatments (Blue: DAPI; Green: TUNEL staining; n=6, **P*<0.05, ****P*< 0.001). Scale bar:100 μm.


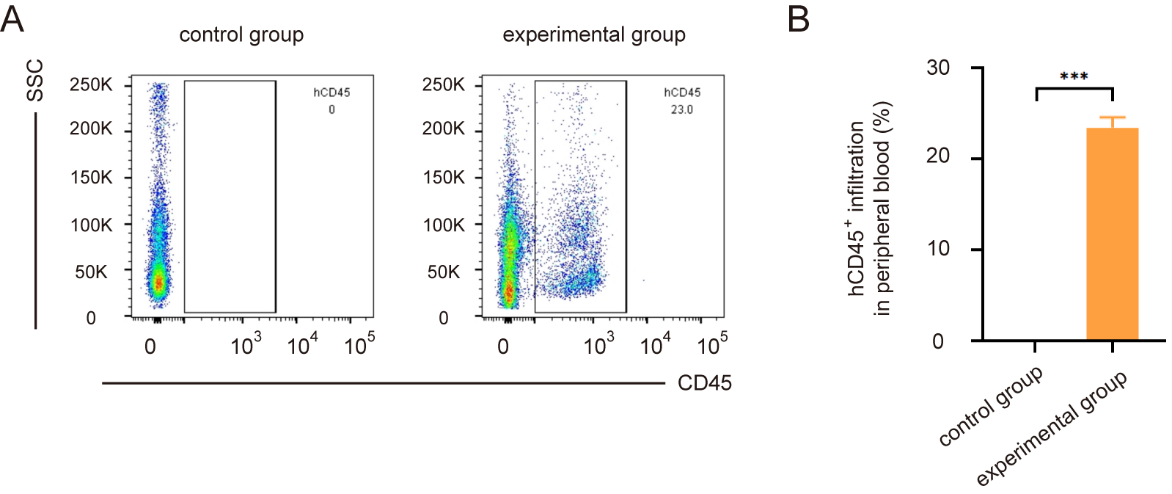


**Figure S11. A)** Representative plots showing human derived immune cells (human CD45 positive cells) in mice peripheral blood analyzed by flow cytometry (control group: saline injection, experimental group: fusion HAC NVs injection). **B)** Statistics analysis of hCD45 positive cells in (A) (n=4, ****P*< 0.001).


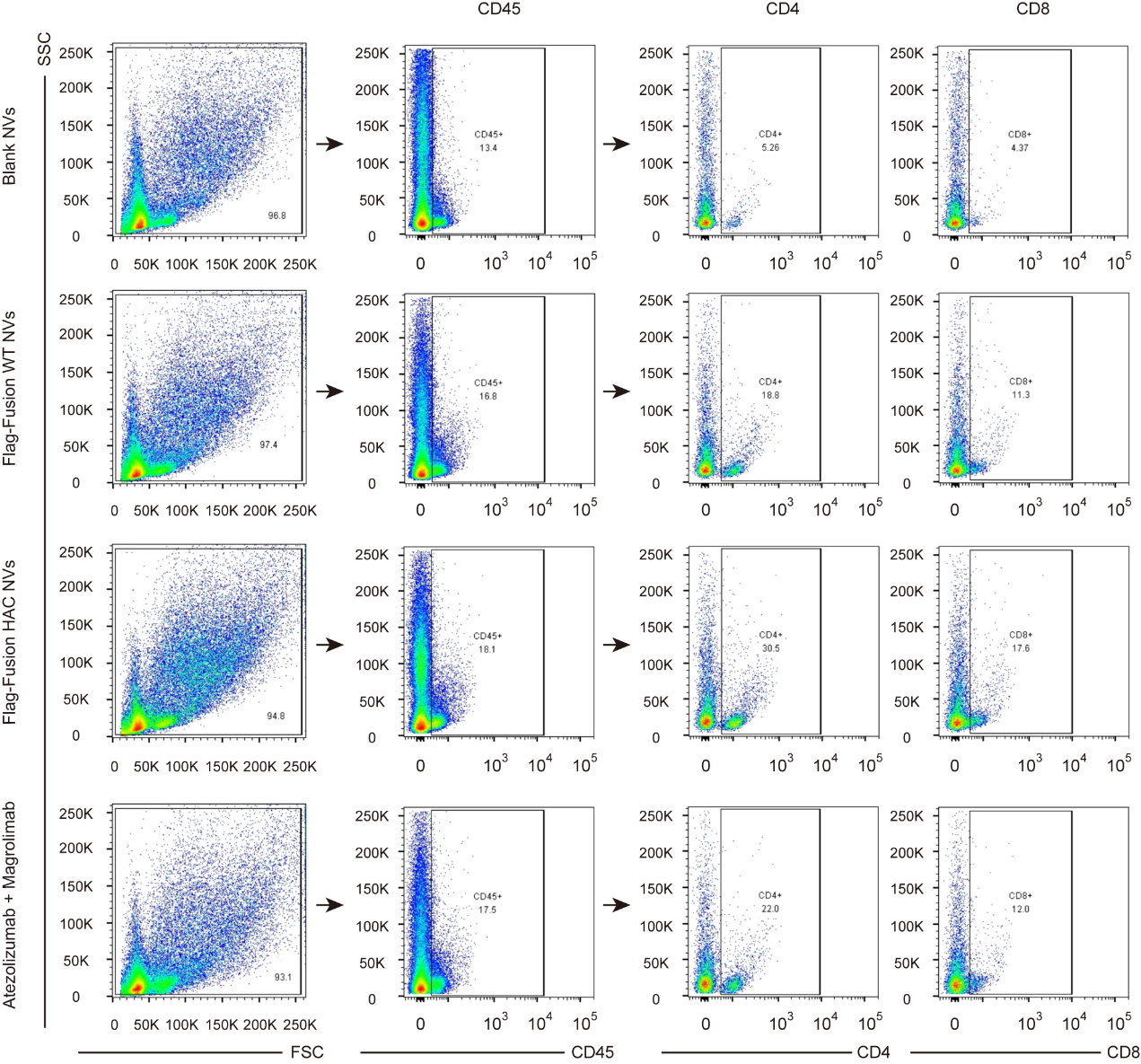


**Figure S12.** Flow cytometry gating strategy for analysis of the percentage of CD45^+^CD4^+^ and CD45^+^CD8^+^ T cells in tumor tissues.


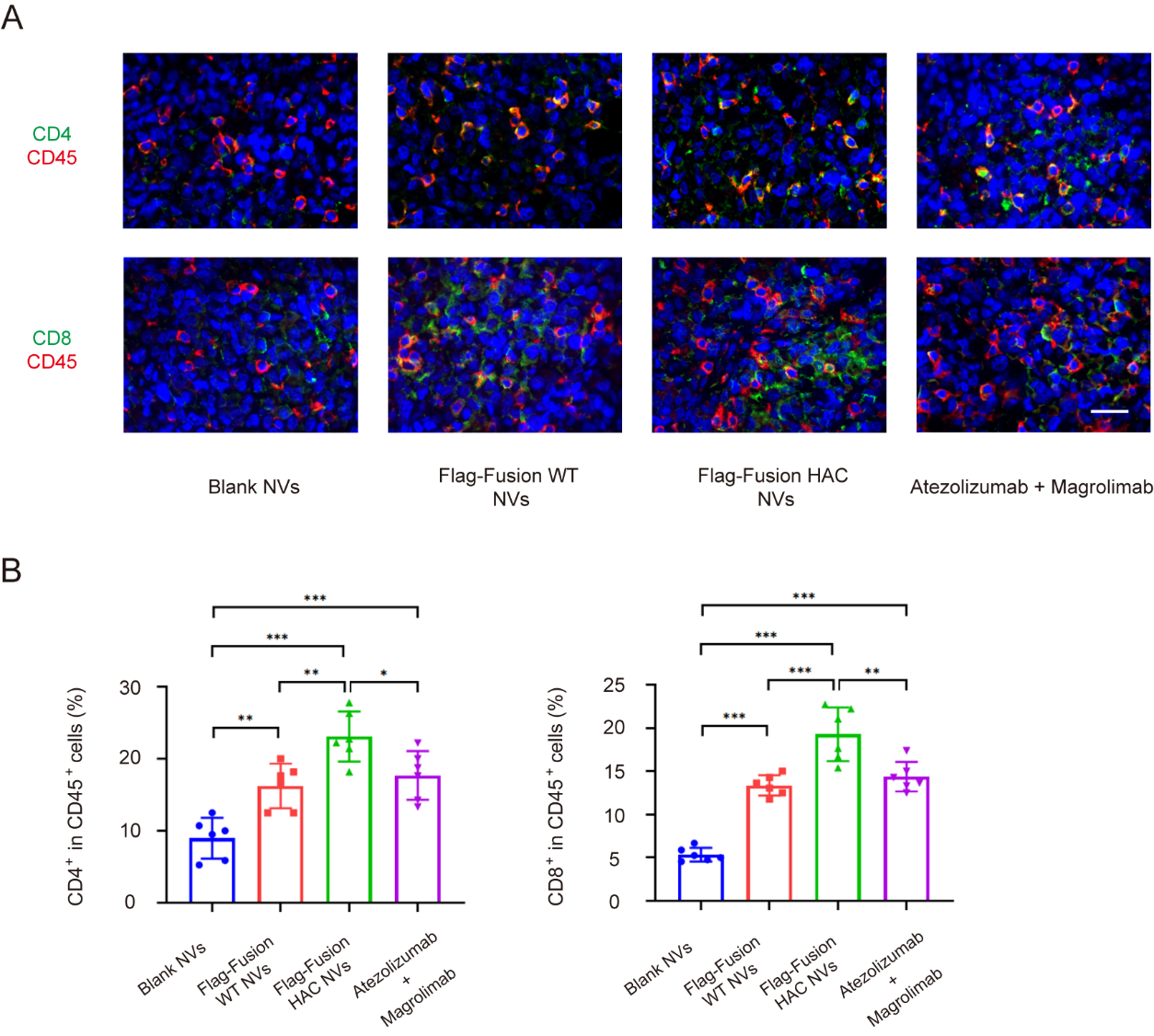


**Figure S13. A)** Representative immunofluorescence images of CD4^+^ and CD8^+^ T cells in tumor tissues after indicated administration (Red: CD45; Green: CD4, CD8; Blue: DAPI). Scale bar: 50 μm. **B)** Statistical analysis of CD4^+^ and CD8^+^ T cells in tumor samples in (A) (n=6, **P*<0.05, ***P*<0.01, ****P*< 0.001).


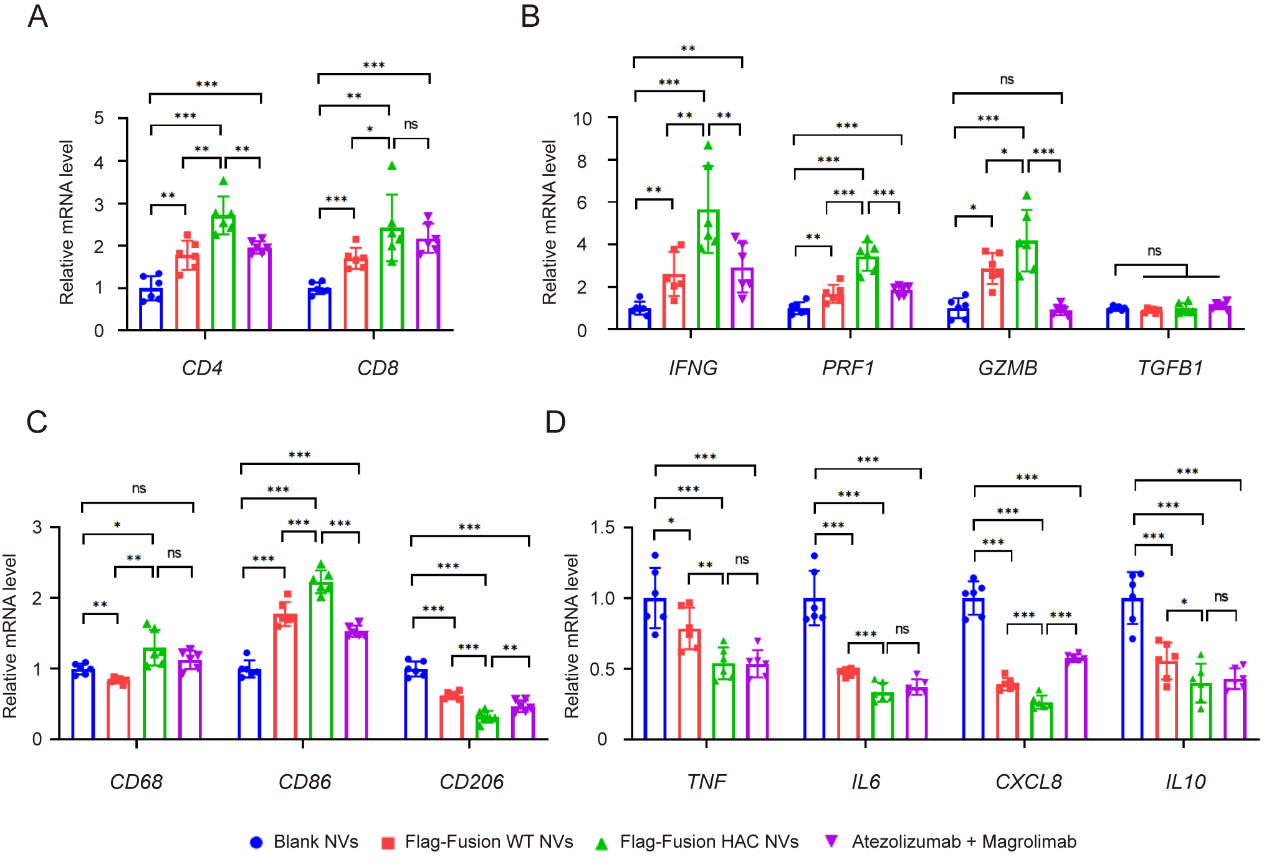


**Figure S14.** Statistics analysis of the mRNA expression of **A)** activated T lymphocyte markers, **B)** cytokines and chemokines produced by activated T lymphocyte, **C)** tumor-associated macrophage (TAM) markers and **D)** cytokines and chemokines produced by TAM were examined by RT-qPCR (n=6, **P*<0.05, ***P*< 0.01, and ****P*< 0.001).


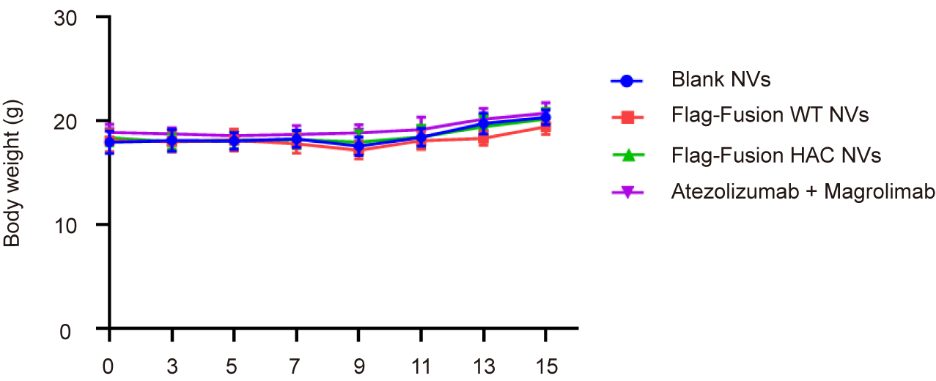


**Figure S15.** Body weight change of mice treated with different treatments (n=6).


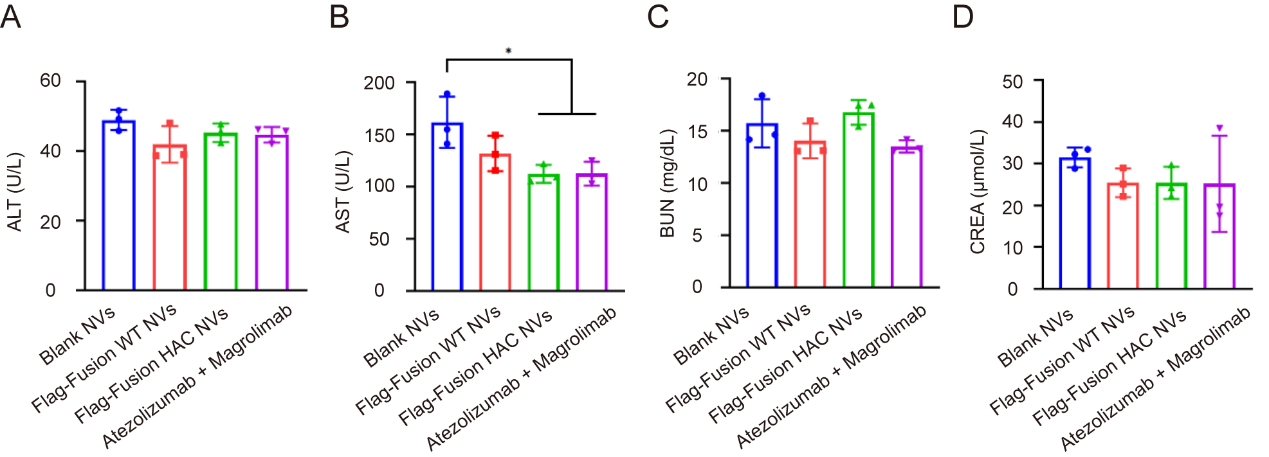


**Figure S16.** The biochemistry level of liver and renal function after different treatments. **A)** alanine transaminase (ALT), **B)** aspartate transaminase (AST), **C)** blood urea nitrogen (BUN) and **D)** creatinine (CREA) (n=3, **P*<0.05).


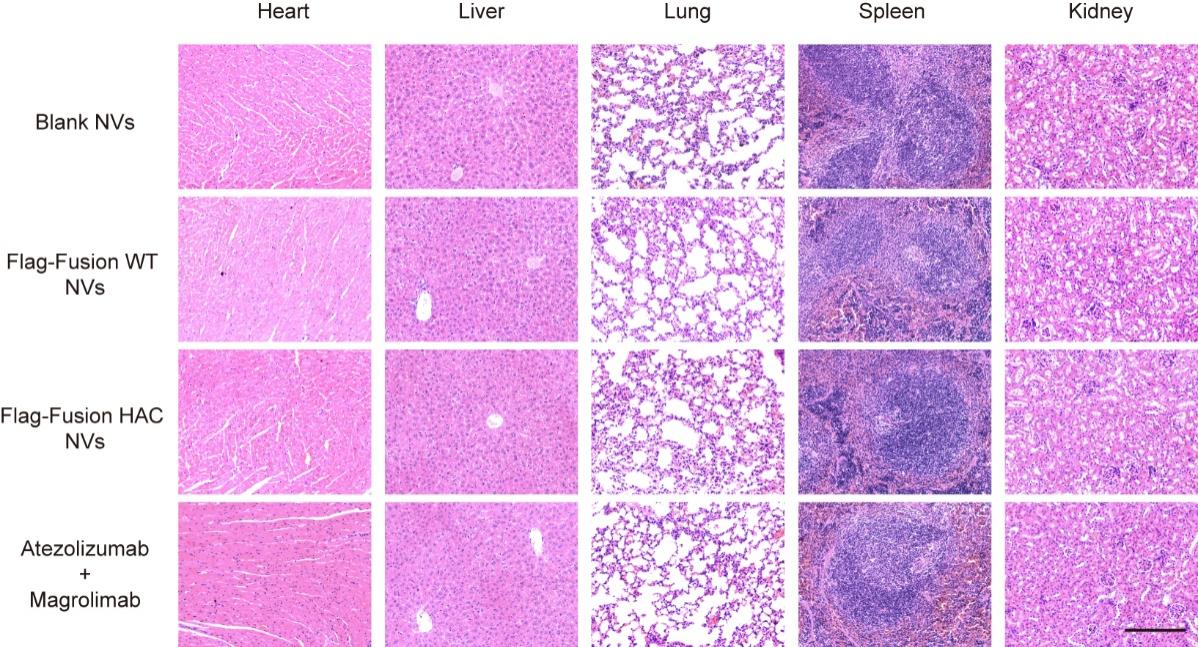


**Figure S17.** H&E staining of the main organs (heart, liver, lung, spleen and kidney) after different treatments. Scale bar:100 μm.

**Reference**

Théry, C., Amigorena, S., Raposo, G., and Clayton, A. (2006). Isolation and Characterization of Exosomes from Cell Culture Supernatants and Biological Fluids. Curr Protoc Cell Biol Chapter 3.

Wen, Y., Chen, Y., Wang, G., Abhange, K., Xue, F., Quinn, Z., Mao, W., and Wan, Y. (2020). Factors influencing the measurement of the secretion rate of extracellular vesicles. The Analyst 145, 5870-5877.
